# A Large-Scale Functional Analysis of Putative Target Genes of Mating-Type Loci Provides Insight into the Regulation of Sexual Development of the Cereal Pathogen *Fusarium graminearum*

**DOI:** 10.1371/journal.pgen.1005486

**Published:** 2015-09-03

**Authors:** Hee-Kyoung Kim, Seong-Mi Jo, Gi-Yong Kim, Da-Woon Kim, Yeon-Ki Kim, Sung-Hwan Yun

**Affiliations:** 1 Department of Medical Biotechnology, Soonchunhyang University, Asan, Chungnam, Republic of Korea; 2 Division of Biosciences and Bioinformatics, Myongji University, Yongin, Gyeonggi-do, Republic of Korea; Christian Albrechts Universitat zu Kiel, GERMANY

## Abstract

*Fusarium graminearum*, the causal agent of Fusarium head blight in cereal crops, produces sexual progeny (ascospore) as an important overwintering and dissemination strategy for completing the disease cycle. This homothallic ascomycetous species does not require a partner for sexual mating; instead, it carries two opposite mating-type (*MAT*) loci in a single nucleus to control sexual development. To gain a comprehensive understanding of the regulation of sexual development in *F*. *graminearum*, we used in-depth and high-throughput analyses to examine the target genes controlled transcriptionally by two-linked *MAT* loci (*MAT1-1*, *MAT1-2*). We hybridized a genome-wide microarray with total RNAs from *F*. *graminearum* mutants that lacked each *MAT* locus individually or together, and overexpressed *MAT1-2-1*, as well as their wild-type progenitor, at an early stage of sexual development. A comparison of the gene expression levels revealed a total of 1,245 differentially expressed genes (DEGs) among all of the mutants examined. Among these, genes involved in metabolism, cell wall organization, cellular response to stimuli, cell adhesion, fertilization, development, chromatin silencing, and signal transduction, were significantly enriched. Protein binding microarray analysis revealed the presence of putative core DNA binding sequences (ATTAAT or ATTGTT) for the HMG (high mobility group)-box motif in the MAT1-2-1 protein. Targeted deletion of 106 DEGs revealed 25 genes that were specifically required for sexual development, most of which were regulated transcriptionally by both the *MAT1-1* and *MAT1-2* loci. Taken together with the expression patterns of key target genes, we propose a regulatory pathway for *MAT-*mediated sexual development, in which both *MAT* loci may be activated by several environmental cues via chromatin remodeling and/or signaling pathways, and then control the expression of at least 1,245 target genes during sexual development via regulatory cascades and/or networks involving several downstream transcription factors and a putative RNA interference pathway.

## Author Summary

The production of sexual propagules via a self-fertile mating strategy in *Fusarium graminearum*, an important cereal pathogen, is essential for overwintering and dissemination during the recurrent disease cycle caused by this fungus. Genome-wide microarray analyses allow the identification of gene sets that are regulated by the mating-type (*MAT*) loci, which is a master regulator of sexual reproduction in *F*. *graminearum*. By employing in-depth and high-throughput functional analyses, the current study provides novel insight into our understanding of the regulation of sexual developmental processes by the *MAT* loci. *MAT* genes, which are located at two linked *MAT* loci, play important roles in even the late stages of sexual development by controlling regulatory pathways involving several sexual-specific transcription factors and putative RNA interference regulators. This study could be significant both practically and fundamentally because of the ecological impact of sexual reproduction by *F*. *graminearum* during disease development in the field.

## Introduction

*Fusarium graminearum*, a homothallic (self-fertile) ascomycetous fungus, causes serious diseases (e.g., Fusarium head blight) in major cereal crops, and produces several mycotoxins in diseased cereals [1]. Recently, this species was defined as a member of the *F*. *graminearum* species complex, which consists of more than 16 phylogenetically distinct species found worldwide [2–7]. To complete the recurrent cycle of cereal diseases, *F*. *graminearum* produces sexual progeny (ascospores) on cereal debris as overwintering propagules [8]. The sexual reproduction of *F*. *graminearum* is controlled by master regulators called mating-type (*MAT*) loci [9, 10]. Unlike their heterothallic relatives, *F*. *graminearum* carries two closely linked *MAT* loci (*MAT1-1*, *MAT1-2*). A single nucleus contains individual *MAT* genes in a structural organization (*MAT1-1-1*, *MAT1-1-2*, *MAT1-1-3* at the *MAT1-1* locus; *MAT1-2-1* at the *MAT1-2* locus) similar to that of other Sordariomycetes (e.g., *Neurospora crassa*, *Podospora anserina*, *Sordaria macrospora*) [9, 10]. All of the *MAT* genes encode transcription factors that carry conserved DNA-binding motifs called an alpha box (*MAT1-1-1*), an HMG-box domain (*MAT1-1-3*, *MAT1-2-1*), and a PHP domain (*MAT1-1-2*) [9, 10].

The importance of individual *MAT* transcripts and *MAT* loci for sexual development has been intensively studied in *F*. *graminearum*, but their functional requirement is not conserved among other fungal species. All of the four individual *MAT* genes at the *MAT* loci are essential for sexual development in *F*. *graminearum* [11–14], whereas *SmtA-1* and *SmtA-3* (comparable to *MAT1-1-1* and *MAT1-1-*3, respectively) are dispensable for fruiting body (perithecium) formation in homothallic *S*. *macrospora* [15]. In heterothallic species, *MAT1-1-2* is essential for perithecium formation in *P*. *anserina*, but has a redundant function together with *MAT1-1-3* in *N*. *crassa* [15]. The phenotypic changes caused by *MAT* deletions and gene expression patterns in *F*. *graminearum* strongly suggest that *MAT* genes are involved in both the early and late stages of sexual development [13, 14]. In contrast, the prominent roles of *MAT* genes in heterothallic species are to maintain the sexual identity of cells that express the opposite *MAT* gene for mating (i.e., controlling sexual compatibility) and to regulate pheromone-mediated signaling pathways. Recently, an additional transcript (*MAT1-2-3*) with no DNA-binding motif was identified in the *MAT1-2* locus [16], but its role(s) in sexual development is not essential in *F*. *graminearum* [14].

MAT transcriptional factors may control the transcriptional expression of downstream genes that are necessary for sexual development in filamentous fungi. Several transcriptional profiling analyses have been performed to identify *MAT* loci target genes that are differentially expressed in fungal strains lacking *MAT* genes during sexual development [17–20]. However, the function and related regulatory pathways of *MAT*-target genes have not been sufficiently elucidated to allow a comprehensive understanding of sexual development under the control of the *MAT* loci; only homology-based functional categorization and limited information regarding gene function (such as pheromone/receptor genes) are available. Very recently, putative target genes of a fungal mating-type gene (*MAT1-1-1*) carrying a DNA binding alpha box domain were identified by a genome-wide search using chromatin immunoprecipitation combined with next-generation sequencing (ChIP-seq) in *Penicillium chrysogenum*, but only a limited number of target genes were functionally characterized [21]. In *F*. *graminearum*, genome-wide transcriptional analyses during perithecium development have been also performed using microarrays [22] and RNA-sequencing (RNA-seq) technology [23], but the functions of most of the highly expressed genes remain unclear.

Despite intensive investigation of *MAT* genes in filamentous fungi, many questions regarding sexual developmental processes regulated by *MAT* genes remain unanswered. Various cellular and developmental events occur during sexual reproduction in ascomycetes: ascogonium formation, fertilization, nuclear migration and proliferation in ascogonium, nuclear recognition and fusion in dikaryotic hyphae, meiosis, and ascus/ascospore formation. However, little is known about the specific roles of *MAT* genes in these sexual stages, particularly those after fertilization, although pheromone/receptor-mediated fertilization under control of *MAT* is well-established in heterothallic species. In homothallic species, the function of *MAT* loci that are present within a single nucleus are less known compared to those in heterothallic species; even the mechanism by which *MAT* controls the mating process remains unclear.

Homothallic *F*. *graminearum* is an ideal species for exploring these unanswered questions for several reasons described below. The presence of both *MAT1-1* and *MAT1-2* loci in a single nucleus provides a good model system for investigating the roles of both loci after fertilization (e.g., nuclear fusion, meiosis, perithecium maturation), which requires two parental strains of the opposite mating types in heterothallic species. The capacity of *F*. *graminearum* to outcross and self-cross [11] suggests that it has gene regulatory mechanisms for sexual development that are identical to those of heterothallic ascomycetes. Unlike *S*. *macrospora*, *F*. *graminearum*, requires all of the transcripts at both *MAT* loci for sexual development [14], which makes the effects of *MAT* deletions on the expression and function of *MAT* target genes more evident. In addition, *F*. *graminearum* can be molecularly manipulated to allow high-throughput gene deletions [24] and genetic analyses [11]. Finally, the production of sexual progeny is ecologically important for disease development by *F*. *graminearum*, because its sexual cycle predominates in the field, which makes the current study significant both practically and fundamentally.

To explore the regulatory mechanisms controlled by *MAT* genes in *F*. *graminearum*, we performed a large-scale study of the target genes of two *MAT* loci using several strategies including genome-wide transcriptional profiling in various genetic backgrounds, protein binding microarray analysis, in-depth quantitative real-time PCR, and high-throughput gene deletions. The results of this study combined with previous reports provide an insight that allows a comprehensive understanding of the sexual developmental processes under the control of the *MAT* loci in *F*. *graminearum*.

## Results

### Identification of differentially expressed genes (DEGs) in each transgenic strain

**Fig 1:**
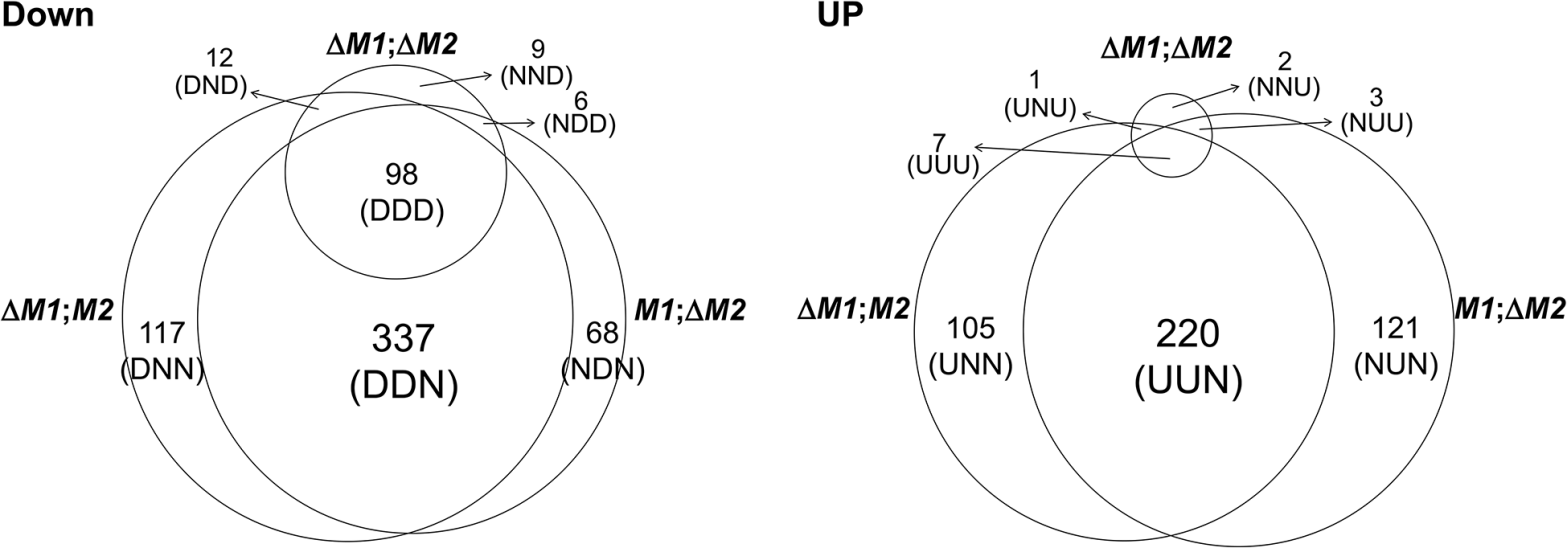
Number of genes expressed differentially in the *F*. *graminearum* strains deleted for the *MAT1-1* locus (0394*M1*;*M2*), *MAT1-2* locus (*M1*; Δ*M2*), and both *MAT1-1* and *MAT1-2* loci (Δ*M1*; Δ *M2*) compared to their wild-type progenitor Z3643. DOWN, downregulated genes; UP, upregulated genes. The numbers of differentially expressed genes (DEGs) in each expression category are indicated above the category designations in parentheses. Abbreviations for DEGs with three characters: U, upregulated, D; downregulated; N, no change. The first, second, and third characters represent the expression pattern in the Δ*MAT1-1*, Δ*MAT1-2*, and Δ*MAT1-1*;Δ*MAT1-2* strains, respectively, compared to the Z3643 strain.

For the microarrays, we used four transgenic strains that were derived from the self-fertile wild-type (WT) strain (Z3643). Three strains, designated Δ*MAT1-1*, Δ*MAT1-2*, and Δ*MAT1-1*;Δ*MAT1-2*, contained different deletions of the two *MAT* loci (*MAT1-1*, *MAT1-2*), and one strain (OM2) overexpressed the *MAT1-2-1* allele (for details, see S1 Text and S1–S3 Figs). To identify genes that were regulated by the *MAT* loci during sexual development, genome-wide microarray analysis was performed using total RNA extracted from mycelia and/or perithecial initials of three *MAT*-deletion strains, OM2, and their WT progenitor (Z3643). Analysis of the transcriptional profiles revealed a total of 1,245 genes that were differentially regulated by ≥ 2-fold in all of the transgenic strains compared to Z3643. Among these, 1,106 (647 downregulated, 459 upregulated) were differentially regulated in the three *MAT-*deletion strains (Δ*MAT1-1*, Δ*MAT1-2*, Δ*MAT1-1;*Δ*MAT1-2*), and 187 (177 downregulated, 10 upregulated) were in OM2 (Fig 1, S1 Table). All of the DEGs identified in the three *MAT*-deletion strains could be categorized into 14 groups according to their expression patterns in each *MAT-*deletion background (Fig 1, S1 Table). Of the 647 genes that were downregulated compared to WT, 522 (80.7%) were in either or both Δ*MAT1-1* and Δ*MAT1-2*, but not in Δ*MAT1-1*;Δ*MAT1-2*. Among these, 337 were downregulated in both Δ*MAT1-1* and Δ*MAT1-2*, but were not significantly changed in Δ*MAT1-1*;Δ*MAT1-2* (designated DDN, where the first D means downregulated in Δ*MAT1-1*, the second D is for the downregulation in Δ*MAT1-2*, and N means no change in Δ*MAT1-1*;Δ*MAT1-2*), 117 were downregulated in only Δ*MAT1-1* (DNN), and 68 were downregulated in only Δ*MAT1-2* (NDN). The remaining 125 genes were downregulated in Δ*MAT1-1*;Δ*MAT1-2*, regardless of the differential regulation in either Δ*MAT1-1* or Δ*MAT1-2*, among which 98 (78.4%) were DDD-type (Fig 1). Among the upregulated genes, most (97.2%) were present in either or both Δ*MAT1-1* and Δ*MAT1-2*, but not in Δ*MAT1-1*;Δ*MAT1-2*; 220, 105, and 121 genes were UUN-, UNN-, and NUN-type, respectively (Fig 1). Most of the DEGs identified in OM2 were downregulated, among which 15 were also downregulated and 30 were also upregulated in the *MAT-*deletion strains (S1 Table). Four of the ten genes upregulated in OM2 were also downregulated in the *MAT-*deletion strains (S1 Table). *MAT1-2-1* was NDD-type and was upregulated in OM2, and two *MAT1-1* transcripts (*MAT1-1-2*, *MAT1-1-3*) were the DND-type, and were unchanged in OM2.

In addition, 729 DEGs (325 downregulated, 404 upregulated) were identified in the two *MAT* deletion strains (Δ*MAT1-1* and Δ*MAT1-2*) compared to the *MAT* null strain (Δ*MAT1-1*;Δ*MAT1-2*) (S4 Fig, S2 Table). In total, 87 of the 101 NNU-type genes (unchanged in Δ*MAT1-1* or Δ*MAT1-2*, but upregulated in WT compared to Δ*MAT1-1*;Δ*MAT1-2*) overlapped with the DDD-type genes that were identified in comparison with WT (S2 Table).

### Functional categorization (annotation) of DEGs

The GO analysis revealed that several Biological Process categories were enriched among the DDN-, DNN-, and NDN-type genes, including various types of metabolism, and developmental processes. Among the genes (UNN, UUN, NUN) upregulated in the *MAT-*deletion strains were enriched the categories of carbohydrate metabolism, developmental processes involved in sporulation, cellular response to chemical stimuli, and cell wall organization. Most of the cellular components categories enriched among these groups were fungal cell wall, plasma membranes, and extracellular (S3 Table). The DDD-type genes were poorly matched to the GO-terms; only those involved in developmental processes (*e*.*g*., regulation of cell morphogenesis and response to stimuli), and organic hydroxyl compound metabolism (including the polyketide biosynthesis for perithecial pigment) categories were enriched in this group (S3 Table). Among the genes that were downregulated in OM2, the categories of metabolism for lipid and nitrogen compounds were enriched. (S3 Table).

In addition to the genes enriched for GO terms, 21 genes that might be involved in the cellular processes (e.g., cell fusion, nuclear fusion, cell division, chromosome partitioning) required for sexual development were identified among the DEGs (S4 Table); the expression of most of these was unchanged in Δ*MAT1*-1;Δ*MAT1-2* (XXN).

### Confirmation of differential gene expression in *MAT*-deletion strains

A total of 58 DEGs identified in this study were analyzed using quantitative real-time PCR in *MAT*-deletion strains at the perithecial induction stage to confirm that their differential expression was caused by each *MAT* deletion. The expression patterns of 42 of the 58 genes compared in the three *MAT*-deletion strains and WT were consistent with the microarray results (S5 Table). The other 16 genes were also differentially expressed, but their patterns in one of three *MAT-*deletion strains were not consistent with the qPCR data. Among these, eight genes that were identified as DDN-type in the microarrays, exhibited expression that was downregulated almost two-fold in Δ*MAT1-1*;Δ*MAT1-2* compared to WT, and were confirmed as DDD-type using qPCR. Previous studies confirmed that an additional eight genes were downregulated in either Δ*MAT1-2* or both Δ*MAT1-1* and Δ*MAT1-2* strains by Northern blot analysis [17, 25].

### Transcription factors among the DEGs

**Fig 2:**
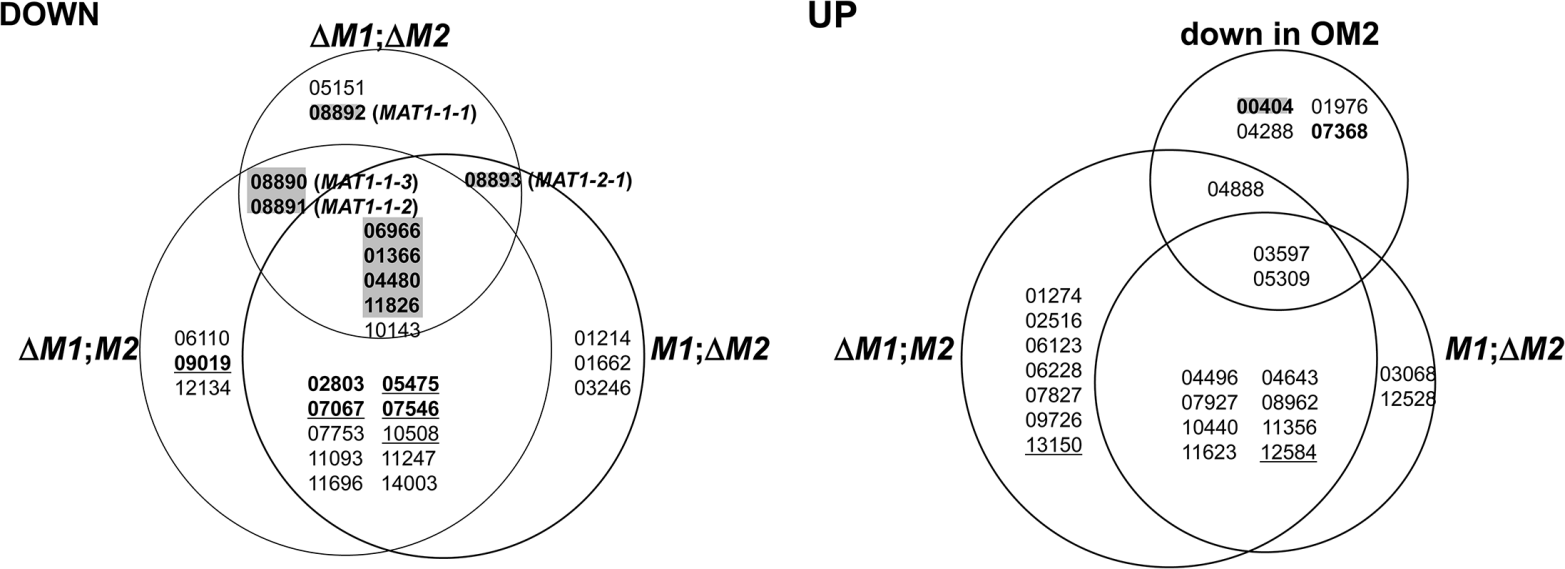
Genes encoding the transcription factors differentially expressed in the *F*. *graminearum* strains deleted for the *MAT1-1* locus (Δ*M1*;*M2*), *MAT1-2* locus (*M1*;Δ*M2*), both *MAT1-1* and *MAT1-2* loci (Δ*M1*;Δ*M2*), and overexpressing *MAT1-2-1* (OM2). DOWN, downregulated genes; UP, upregulated genes. The gene expression categories in the Venn diagrams are the same as those in Fig 1. The numbers in each category indicate the gene ID (FGSG ID) in the *F*. *graminearum* genome database.

A total of 50 transcription factor (TF) genes (6.9% of the total TFs in the *F*. *graminearum* genome) [24] were identified among the DEGs in the current study (Fig 2). TF genes with a specific and essential function for sexual development in *F*. *graminearum*, which were identified based on phenotypic changes after gene deletions [24], were only enriched among gene groups that were downregulated in Δ*MAT1-1*;Δ*MAT1-2* (DND, NDD, NND, DDD). All of the sexual development-specific TFs, other than the *MAT* genes themselves, were DDD-type. The TFs belonging to other gene groups, whose expression levels were unchanged in Δ*MAT1-1*;Δ*MAT1-2* (DDN, DNN, NDN), were either dispensable for sexual development (ten TFs), involved in pleiotropic phenotypes (i.e. involved in sexual development and other traits; four TFs), or involved in traits other than sexual development (two TFs; Fig 2). In contrast, none of the TFs that were specific to sexual development were identified among the 20 TFs upregulated in the *MAT*-deletion strains; only 1 TF gene deletion proved lethal. Among the four TFs downregulated in OM2, only one TF (FGSG_00404) was sexual-specific in the Z3639 strain in a previous study [24], but was not in Z3643 in the current study. The remaining TFs were dispensable or were involved in sexual development along with other trait (zearalenone production) (FGSG_07368) (Fig 2).

Based on the phenotypic changes by gene deletions [23], the *F*. *graminearum* locus IDs (FGSG_) on a gray background indicate the genes specific in function to sexual development, IDs in bold and underline are for those involved in sexual development and other traits, IDs in bold are for those are involved in the traits other than sexual development, and underlined IDs are for those probably lethal.

### Identification of gene clusters for secondary metabolites (SMs) among the DEGs

We focused on the expression profiling of genes involved in secondary metabolism since it has been known that secondary metabolism and sexual development are linked in filamentous fungi. To identify SM genes among the DEGs, they were compared against members of the 67 tentative SM gene clusters in *F*. *graminearum* [26]. Gene member(s) belonging to 22 SM clusters were identified in the DEGs from the *MAT-*deletion strains or OM2 (S6 Table); however, only 6 SM clusters included DEGs that encoded key (signature) enzymes. Among these, members of two polyketide synthase (*PKS*) gene clusters were downregulated in the *MAT*-deletion strains: *PKS3* (along with four additional genes), which is responsible for the biosynthesis of dark perithecial pigment, and *PKS7* (with one tailoring gene), whose chemical product has not yet been identified; these were DDD- and DDN-type, respectively. Two non-ribosomal peptide synthetase genes (*NPS10*, a *NPS-*like gene) for unknown metabolites were also identified. In addition to these key enzyme genes, those that encoded either tailoring enzymes or transporters belonging to other *PKS* clusters (*PKS2*, *PKS14*, *PKS15*, *PKS17* for unknown polyketide compounds), an *NPS1* cluster for a siderophore (malonichrome) [27], and a butenolide cluster were identified among the DEGs (S6 Table).

### Expression of genes identified previously in *F*. *graminearum* among the DEGs

A total of 169 DEGs (13.6%) identified in this study overlapped with the 2,064 genes identified previously as sexual development-specific in the *F*. *graminearum* PH-1 strain, and whose transcripts were only detected during perithecium formation [22]. The genes downregulated in Δ*MAT1-1*;Δ*MAT1-2* (*i*.*e*. NND-, NDD-, DND-, DDD-type) overlapped at a higher frequency (43 of 125; 34.4%) than those downregulated in both or either Δ*MAT1-1* and Δ*MAT1-2* (DDN, DNN, NDN) (76 of 522; 14.6%) (S7 Table).

**Fig 3:**
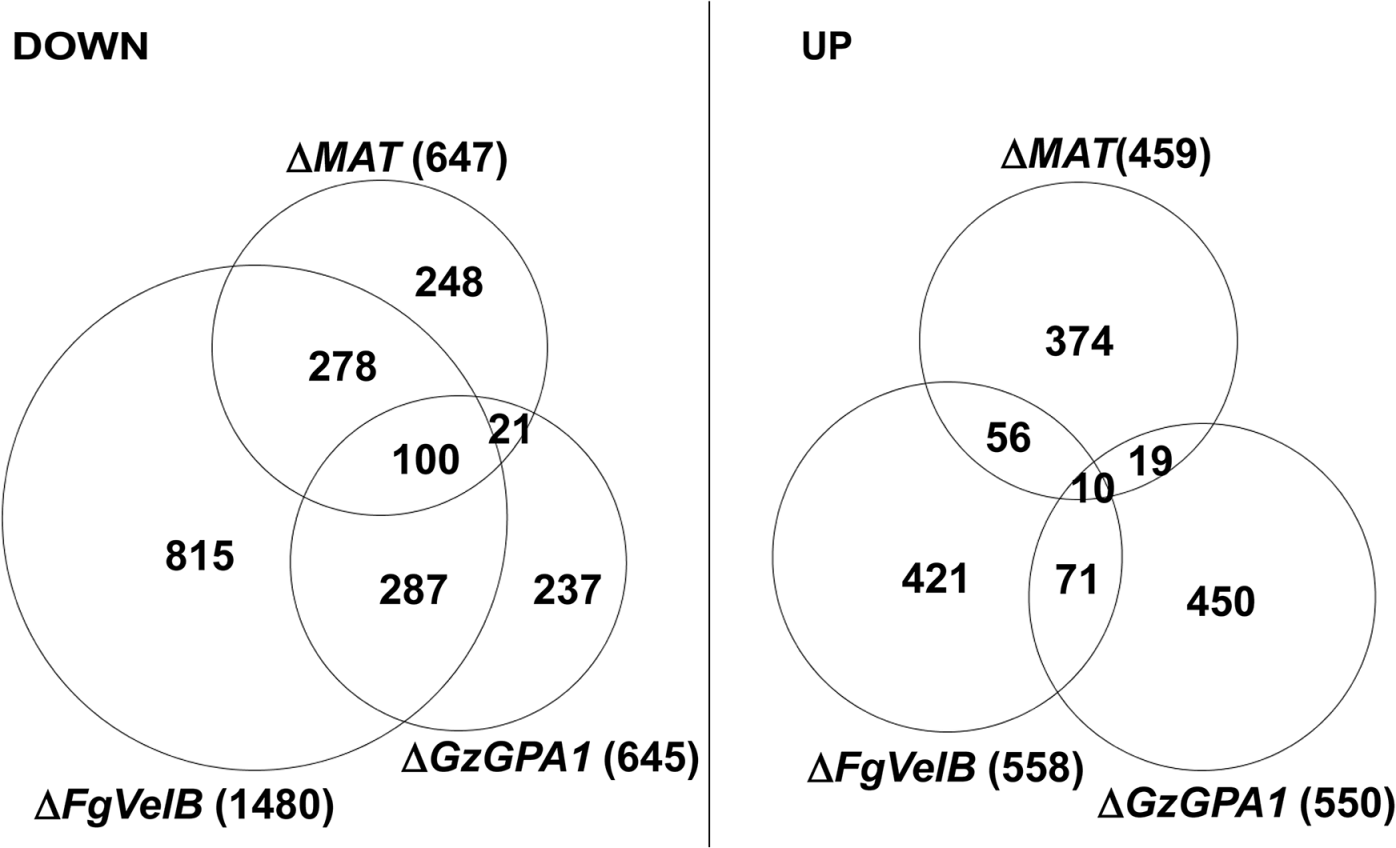
Number of DEGs in the *F*. *graminearum* strains lacking the individual *MAT* or both *MAT* loci (Δ*MAT*) overlapped with those from the Δ*FgVelB* and Δ*GzGPA1* strains, respectively. DOWN, downregulated genes; UP, upregulated genes. The numbers of total DEGs in each category are indicated in parentheses next to the fungal strains.

The DEGs identified in this study were also compared to those from the *F*. *graminearum* strains lacking *FgVelB* or *GzGPA1*, both of which are self-sterile [28, 29]. More than half of the genes (378 of 647; 58.4%) that were downregulated in the *MAT*-deletion strains overlapped with those in *F*. *graminearum* Δ*FgVelB* (Fig 3, S8 Table). In addition, 121 genes (18.7%) downregulated in the *MAT*-deletion strains overlapped with those in the Δ*GzGPA1* strain, among which 100 (82.6%) were also DEGs in the Δ*FgVelB* strain (Fig 3, S8 Table). Interestingly three *MAT* genes [*MAT1-1-*3 (FGSG_08890), *MAT1-1-2* (FGSG_08891), and *MAT1-2-1* (FGSG_08893)], and one *MAT* gene [*MAT1-1-3* (FGSG_08890)] were downregulated in the Δ*FgVelB* [28] and Δ*GzGPA1* [29] strains, respectively.

Surprisingly, only a small number of DEGs overlapped with DEGs in *S*. *macrospora* strains lacking *MAT* genes. Specifically, 19 DEGs overlapped with 311 genes regulated exclusively in Δ*SmtA-2* (Δ*MAT1-1-2*), 26 DEGs corresponded to 520 genes regulated in both Δ*SmtA-1* and Δ*SmtA-2* [15], and 6 DEGs overlapped with 80 genes from Δ*Smta-1* (Δ*MAT1-2*) [19] (S9 Table).

### Transcriptional expression of genes encoding pheromone precursors and receptors

To assess how the *MAT* loci regulate the expression of genes encoding pheromones (GzPPG1, GzPPG2) and their cognate receptors (GzPRE1, GzPRE2) during the early stage of perithecial induction (3 days after the removal of the aerial mycelia on carrot agar), qPCR was used to compare the transcript levels of each gene in the fungal strains used in microarray analysis, as well as in those lacking individual genes (*MAT1-1-1*, *MAT1-1-2*, *MAT1-1-3*) in the *MAT1-1* locus (Table 1). Because northern blotting previously confirmed that all four genes were only expressed in the WT strain during sexual development [30], we used the transcript level of each gene in WT, or the weakest expression level in *GzPRE1* as references to evaluate the effects of *MAT* deletion or overexpression (Table 1). The expression of *GzPPG1* was significantly reduced in all of the *MAT-*deletion strains examined compared to WT, with the exception of Δ*MAT1-1-2*. Because of the relatively high abundance of the *GzPPG1* transcript compared to other genes in WT, this suggests that *GzPPG1* is highly expressed only in the WT and Δ*MAT1-1-2* strains, but not in a *MAT-*locus-specific manner. In contrast, *GzPPG2* expression was reduced in the Δ*MAT1-2* and *MAT*-null strains, but increased dramatically in the strain lacking the entire *MAT1-1* locus (Δ*MAT1-1*), and that lacking only the *MAT1-1-1* gene at the *MAT1-1* locus. This suggests that *GzPPG2* is only expressed in the WT and Δ*MAT1-1* strains, and therefore exhibits a *MAT1-2*-locus-specific expression pattern, consistent with our previous study [30]. *GzPRE1* was downregulated in both the Δ*MAT1-1* and *MAT-*null strains, but was expressed at comparable levels in the Δ*MAT1-2* and WT strains, suggesting a *MAT1-1-*locus-specific expression. However, the upregulation of *GzPRE1* in strains lacking individual *MAT1-1* transcripts was surprising, although most of the upregulated transcript were expressed at levels that were weaker than or similar to *GzPRE2* in WT. In contrast, *GzPRE2* was upregulated in all of the *MAT*-deletion strains except for Δ*MAT1-2*, suggesting that *GzPRE2* was constitutively expressed in all of the strains examined. The effects of Δ*MAT1-1-1* on the expression of genes encoding pheromones and their receptors were more dramatic than those of other *MAT1-1* gene deletions (Δ*MAT1-1-2*, Δ*MAT1-1-3*), with the exception of *GzPRE1*, suggesting that *MAT1-1-1* is the major regulator of the pheromone/receptor system among the three transcripts at the *MAT1-1* locus. In addition, the expression of *GzPPG2*, *GzPRE1*, and *GzPRE2* was upregulated in the OM2 strain (Table 1).

**Table 1:** Relative transcript levels of genes for pheromone precursors and receptors accumulated in the *MAT-*deletion- and *MAT1-2-1-*overexprssing-strains of *F*. *graminearum* under perithecial induction stage.

Gene	Fungal strains							
	WT^a^	Δ*MAT1-1-1* ^b^	Δ*MAT1-1-2* ^c^	Δ*MAT1-1-3* ^d^	Δ*MAT1-1* ^e^	Δ*MAT1-2* ^f^	Δ*MAT1-1*; Δ*MAT1-2* ^g^	OM2^h^
*GzPPG1* ^i^	1.00±0.0	**0.0**±**0.0**	0.8±0.3	**0.3**±**0.1**	**0.0**±**0.0**	**0.0**±**0.0**	**0.0**±**0.0**	1.2±0.3
*GzPPG2* ^i^	1.00±0.0	**91.5**±**6.3**	1.0±0.1	0.5±0.0	**10.0**±**0.1**	**0.0**±**0.0**	**0.0**±**0.0**	**36.8**±**8.4**
*GzPRE1* ^i^	1.00±0.0	**2.5±2.6**	**10.3±4.9**	**5.3±0.7**	**0.3±0.2**	0.7**±**0.4	**0.3±0.4**	**12.4±0.7**
*GzPRE2* ^i^	1.00**±**0.0	**62.9±11.6**	**3.2±1.5**	**3.0±1.0**	**5.0±2.0**	1.7**±**0.5	**2.4±1.2**	**10.2±0.3**
*GzPPG1* ^j^	**1669.0±93.7**	1.0**±**1.4	**1395.1±167.2**	**572.2±64.8**	**0.4±0.2**	0.8±0.3	**0.3±0.4**	**1952.3±41.2**
*GzPPG2* ^j^	**98.0±13.7**	**8961.1±310.5**	**98.2±25.6**	**51.2±6.0**	**976.6±52.2**	**4.0±1.6**	**2.4±1.3**	**3608.1±76.2**
*GzPRE1* ^j^	1.0±0.0	**2.5±2.6**	**10.3±4.9**	**5.3±0.7**	**0.3±0.2**	0.7±0.4	**0.3±0.4**	**12.4±0.7**
*GzPRE2* ^j^	**4.7±1.5**	**295.3±37.1**	**14.9±1.7**	**14.3±3.8**	**23.4±3.8**	**7.9±2.4**	**11.1±0.4**	**47.8±1.0**

Numbers represent the average relative amount of each gene transcript with standard deviation. The numbers in bold were significantly different from the reference data according to Tukey’s test (P < 0.05).

^a^ *F*. *graminearum* Z3643 strain.

^b^ *F*. *graminearum* Z3643 strain lacking the *MAT1-1-1* transcript at the *MAT1-1* locus [14].

^c^ *F*. *graminearum* Z3643 strain lacking the *MAT1-1-2* transcript at the *MAT1-1* locus [14].

^d^ *F*. *graminearum* Z3643 strain lacking the *MAT1-1-3* transcript at the *MAT1-1* locus [14].

^e^ *F*. *graminearum* Z3643 strain lacking all of three transcripts at the *MAT1-1* locus (T43ΔM1-3) [11].

^f^ *F*. *graminearum* Z3643 strain lacking *MAT1-2-1* at the *MAT1-2* locus (T43ΔM2-2) [11].

^g^ *F*. *graminearum* Z3643 strain lacking both *MAT1-1* and *MAT1-2* loci (T43ΔM1M2) generated in this study.

^h^ *F*. *graminearum* Z3643 strain overexpressing *MAT1-2-1*, generated in this study.

^i^The data were obtained by using the transcript level of each gene from WT was used as a reference.

^j^The data were obtained by using the transcript level of *GzPRE1* from WT was used as a reference.

Recently, similar gene expression data for pheromone/receptor genes in the *MAT-*deletion strains were reported by Zheng et al [13]. However, those data cannot be directly compared with those in the current study because they were obtained using RNA samples from aerial hyphae on carrot agar before perithecial induction [13].

### Functional characterization of the selected DEGs

To determine the functional requirement of the DEGs identified in the current study during sexual development, we selected 106 DEGs based on their expression patterns and putative functional roles. Then, we deleted each DEG from the *F*. *graminearum* Z3643 genome using a targeted gene replacement strategy. Including the 32 DEGs that overlapped with those previously identified as being functionally required for or transcriptionally specific for sexual development and/or other traits (e.g., hyphal growth, toxin production, virulence) in *F*. *graminearum*, the results of the functional analysis of a total of 127 DEGs were reported here (S10 Table, S11 Table, Table 2). Based on the phenotypes of the gene deletion strains, 40 genes were responsible for phenotypic changes. Among these, 37 were involved in sexual development alone or together with other traits, and the remaining three were required for phenotypes other than sexual development (S10 Table). Of the 37 genes involved in sexual development, 25 genes were specific to sexual development (Table 2). The phenotypic changes caused by the deletion of these genes were restricted to only sexual developmental processes ranging from the formation of perithecium initials to ascospore discharge; no changes in other traits such as hyphal growth and pigmentation, conidiation, mycotoxin production, and/or virulence were observed. The transgenic strains in which five genes had been individually deleted (FGSG_00404, 04480, 05239, 13708, and 03916) produced no perithecium initials on carrot agar, and those in which each of nine genes were deleted (FGSG_01366, 08320, 11826, 13162, 10742, 08890 [*MAT1-1-3*], 08891 [*MAT1-1-2*], 08892 [*MAT1-1-1*], 08893 [*MAT1-2-1*]) produced barren perithecia that were smaller in size and/or number than WT and contained no asci/ascospores (S11 Table, Table 2, S5 Fig). By contrast, the remaining 11 genes were not absolutely required for the production of fertile perithecia, but instead, were specifically involved in sexual development. The mutants in which six genes (FGSG_03673, 05151, 07578, 06059, 11962, and 02655 [*GzPRE2*]) were deleted individually produced lower numbers of mature perithecia, whereas those lacking FGSG_06966 and FGSG_01862 produced larger perithecia, or showed delayed perithecia formation, respectively, compared to WT. The deletion mutants of FGSG_00348, FGSG_02052, and FGSG_09182 (*PKS3*) produced perithecia that looked similar to those in WT, but exhibited defects at different stages of perithecia maturation (Table 2, S5 Fig).

**Table 2:** DEGs required for sexual development in *F. graminearum*.

Cellular event^a^	Perithecium development^a^	Protein	Proposed function	Phenotype^*^ by gene deletion^b^	Expression category	
	**Mating**					
	**FGSG_02655**	GzPRE2	pheromone receptor	NC or fewer P		U in OM2
Cell aggregation	**FGSG_08890**	MAT1-1-3		smaller P, no AS/AP		DDD^**^
	**FGSG_08891**	MAT1-1-2		smaller P, no AS/AP		DDD^**^
	**FGSG_08892**	MAT1-1-1		smaller P, no AS/AP		DDD^**^
	**FGSG_08893**	MAT1-2-1		smaller P, no AS/AP		DDD^**^
	**Ascogonium (perithecium initials) formation**					
	**FGSG_00404**	TF	Regulation	no P in Z39, but NC in Z43		D in OM
Cell fusion	**FGSG_04480**	TF	Regulation	no P		DDD
	**FGSG_05239**	G protein coupled receptor	Regulation	no P		DDN
Cell adhesion	**FGSG_13708**	*O-*methylase	Metabolism	no P		DDD^**^
	**FGSG_03916**	Fibronectin-attachment protein	Cell adhesion	no P		NUN
	FGSG_06039	Acyl citrate lyase 2	Metabolism	no P reduced C,V, T		DNN
	FGSG_09896	Isocitrate lyase 1	Metabolism	no P, albino aerial mycelia		D in OM2
	FGSG_10825	homocysteine transferase	Metabolism	no P, no aerial mycelia, reduced V, T		DDD^**^
	**Perithecium wall and paraphyses formation**					
	**FGSG_01366**	TF	Regulation	smaller P, no AS		DDD
	**FGSG_08320**	Cytochrome P450	Secondary metabolism	smaller P, no AS		DDD
Polarity	**FGSG_11826**	TF	Regulation	smaller P, no AS		DDD^**^
	**FGSG_13162**	Histone promoter control 2	Chromatin silencing	smaller P, no AS		DDD
	**FGSG_10742**	Pheromone-regulated membrane protein	Cellular fusion	delayed PM, smaller P, no AS		DDD
Programed cell death	FGSG_06228	TF, RGS	Regulation	smaller P, no AS, fewer C, no V, more T in Z39, but fewer C (~11-fold ↓) only in Z43		UNN
	**FGSG_09182**	PKS3	Perithecial wall pigmentation	albino P		DDD
	FGSG_08795	PKS7	Secondary metabolism	smaller P, no AS, abnormal C		DDN
	**FGSG_03673**	Zn-carboxypeptidase	Metabolism	fewer P, no AS		DDD^**^
	**FGSG_05151**	TF	Regulation	fewer P		NND
	**FGSG_07578**	3-dehydroquinate synthetase	Metabolism	fewer P, no AS		DDD
	FGSG_07869	Short-chain dehydrogenases	Metabolism	fewer P, no AS,		DDD
Meiosis	FGSG_09019	TF carrying homeodomain	Regulation	fewer P, VG, T, no V		DNN
	**Ascus development**					
	**FGSG_00348**	Argonaute-like protein	RNA interference	fewer AS/AP		DDD
	**Spore formation and perithecium maturation**					
	FGSG_00532	Vesicle coat complex COPII	Intracellular trafficking & secretion	more P, more P pigmentation, but fewer AS/AP		DDN
	FGSG_07546	TF, MYT2	Regulation	larger P, faster growth, moreaerial mycelia, more AP, reduced V		DDN
	FGSG_09834	Pyruvate decarboxylase	Regulation	no AS, reduced lipidaccumulation in aerial mycelia		D in OM2
	**FGSG_06966**	TF	Regulation	delayed PM in Z39, but larger P in Z43		DDD^**^
	**FGSG_06059**	GAL10-UDP-glucose 4-epimerase	Metabolism	fewer P		DDD
	**FGSG_01862**	Microtubule associated protein	Cytoskeleton dynamics	delayed PM		DDD^**^
	**FGSG_11962**	Hypothetical protein	NA	fewer P		DDD
	FGSG_07368	TF	Regulation	fewer P, ZEA overproduction (~3-fold ↑)		D in OM2
	FGSG_02572	Universal stress protein		fewer P, reduced V in Z39, but NC in Z43		UNN
	**Spore firing**					
	**FGSG_02052**	Hypothetical protein	NA	faster cirrhi production		DDD

^*^Refer to S5 Fig for quantitative comparisons between the wild type and gene-deletion strains showing the phenotypic changes in size or number of perithecia.

^a^Refer to [40].

^b^The *F*. *graminearum* Z3643 (Z43) strain was a wild-type strain for the gene deletions. The other strain Z3639 (Z39) was specifically indicated when used in gene deletion.

Genes (FGSG_) in bold are specific to sexual development.

AP: ascospore, AS: asci, C: conidiation, H: hyphal growth, NA: not assigned, NC: no change, P: perithecia, PM: perithecia maturation, RGS: Regulator of G-protein signaling, SD:sexual development, T: mycotoxin production, V: virulence, VG: vegetative growth, ZEA: zearalenone.

↑: increased

↓: reduced.

^**^determined by qPCR

**Fig 4:**
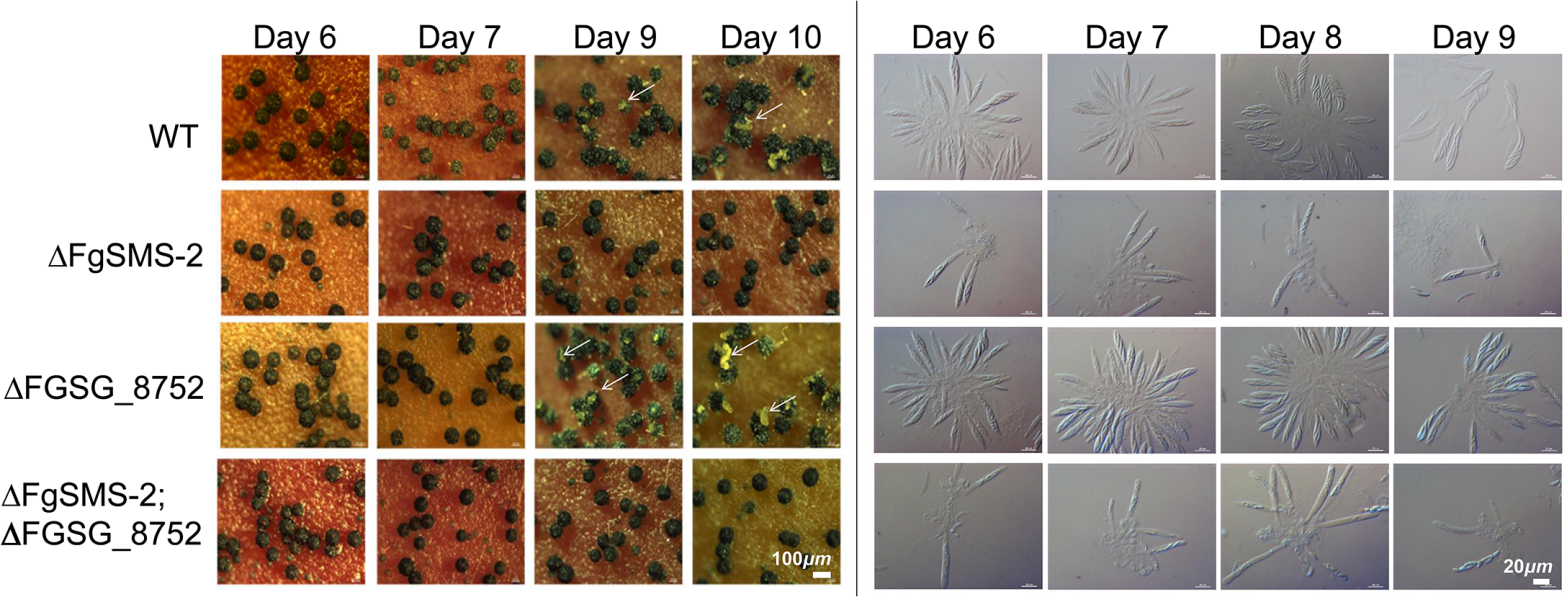
Perithecia formation and ascus/ascopore development in the single gene deletion- (Δ*FgSMS-2* and ΔFGSG_08752, respectively) and double gene deletion-strains (Δ*FgSMS-2*;ΔFGSG_8752) grown on carrot agar. WT: *F*. *graminearum* wild-type Z3643 strain. The perithecia formed 6, 7, 9, or 10 days after perithecia induction, and the asci and ascospores that developed within a perithecium were observed through a dissecting microscope (left panels) and differential interference contrast microscope (right panels), respectively. The exuded spore masses called cirrhi from the perithecia in the wild-type and ΔFGSG_08752 strains were indicated by arrows. Scale bars = 100 μm for perithecia and 20 μm for asci/ascospores.

**Fig 5:**
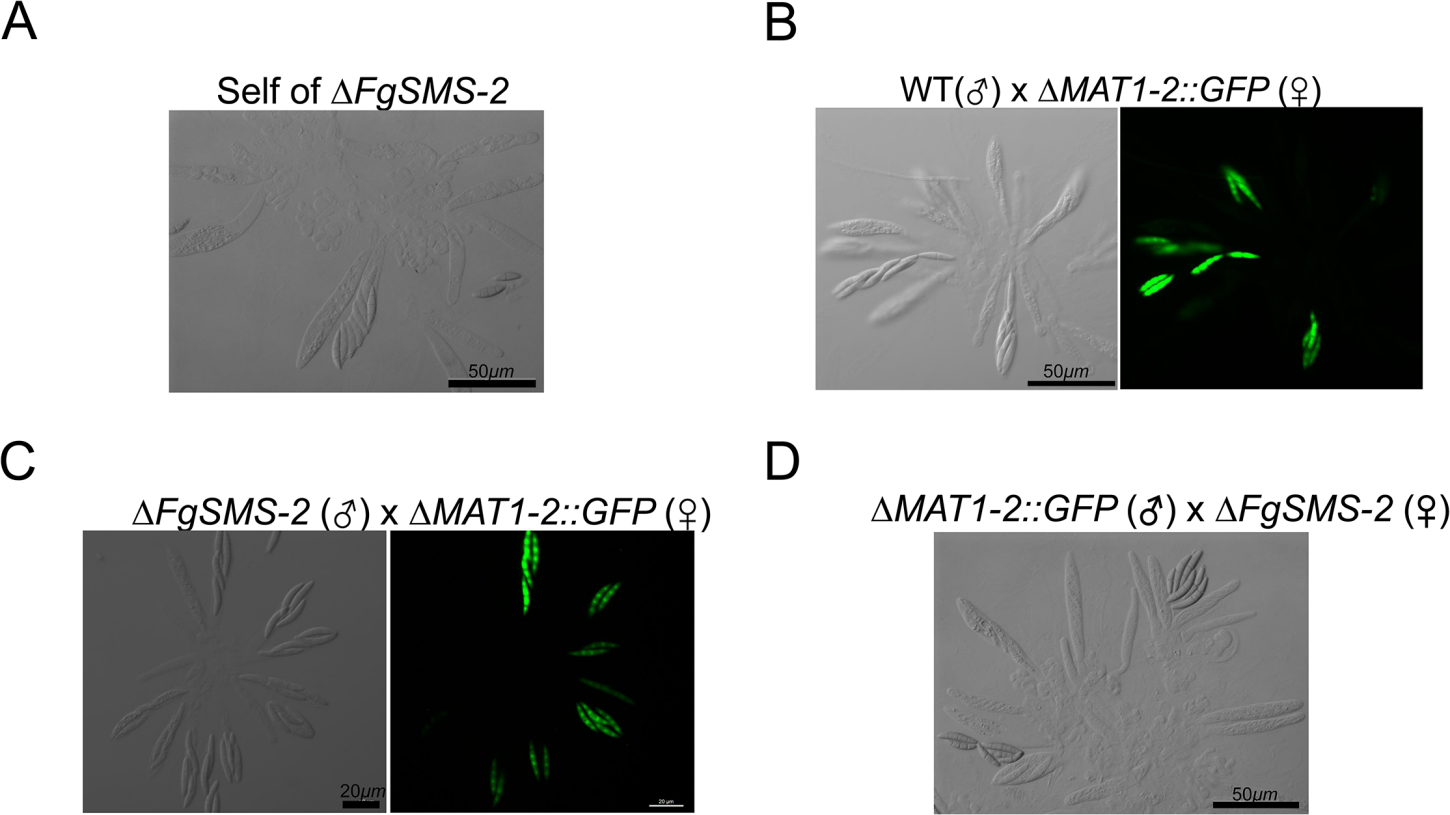
Morphology of asci and ascospores produced in the self-cross of Δ*FgSMS-2* (A) and outcrosses between WT(♂) and Δ*MAT1-2*::*GFP*(♀) (B), between Δ*FgSMS-2*(♂) and Δ*MAT1-2*::*GFP*(♀) (C), and between Δ*MAT1-2*::*GFP*(♂) and Δ*FgSMS-2*(♀) (D). WT, the self-fertile *F*. *graminearum* wild-type Z3643 strain; Δ*FgSMS-2*, a transgenic Z3643 strain lacking the *FgSMS-2* gene; Δ*MAT1-2*::*GFP*, a self-sterile transgenic Z3643 strain where the *MAT1-2* locus was replaced with a green fluorescence protein gene (*GFP*). For the outcrosses, the mycelia of strain acting as a female (♀) were spermatized with conidia of a male-acting strain (♂), as described previously [11]. Only four out of eight ascospores in each ascus fluoresced, as observed by confocal laser microscopy in the outcross between wild-type and Δ*MAT2*::*GFP* (B), whereas the outcross of Δ*FgSMS-2* to Δ*MAT1-2*::*GFP* (C) produced incomplete tetrads, in which the *GFP* marker did not segregate equally.

**Fig 6:**
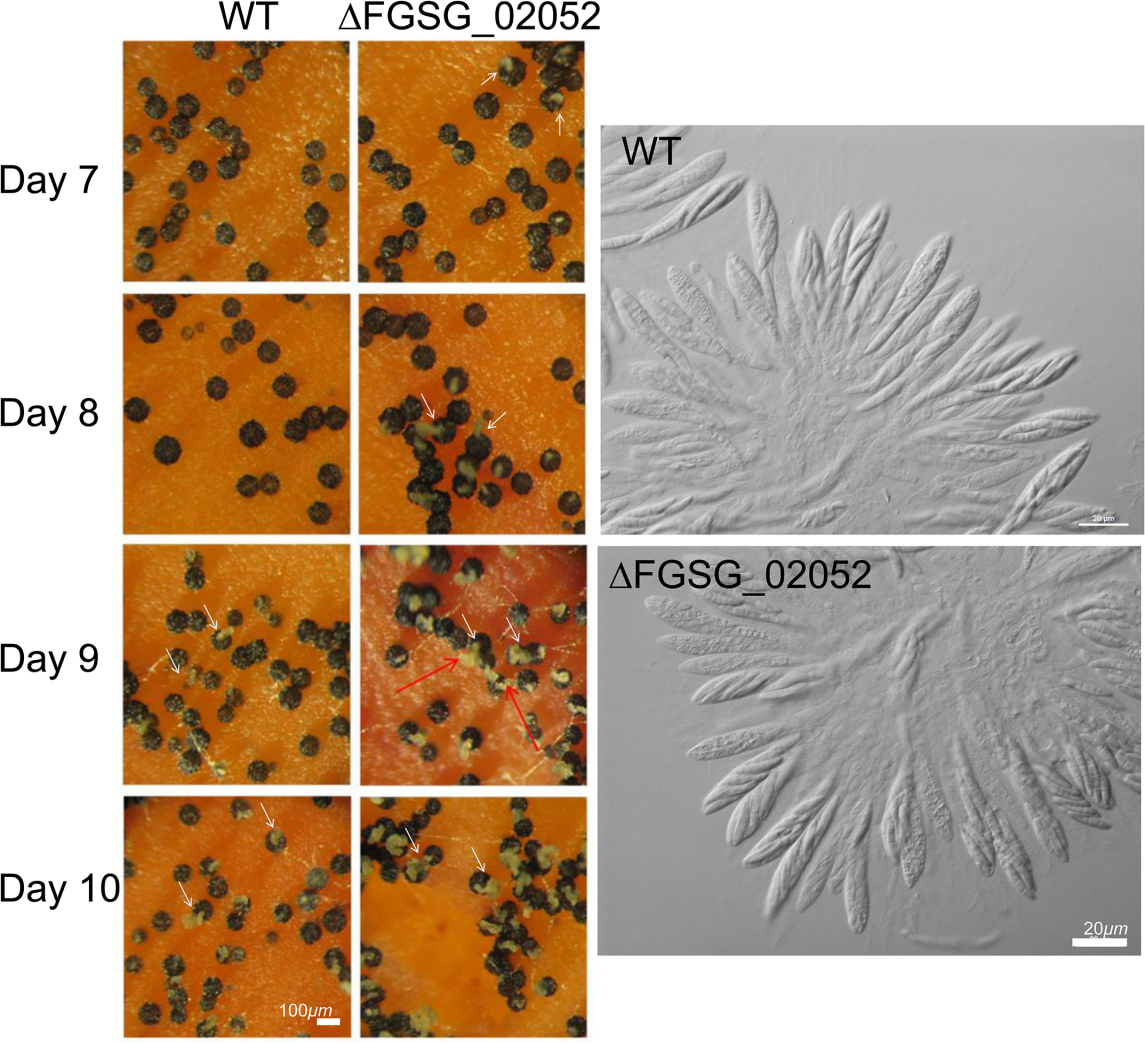
Formation of perithecia (left panels) and the morphology of asci and ascospores within a perithecium in the *F*. *graminearum* deleted for FGSG_02052 (ΔFGSG_02052) grown on carrot agar. WT, *F*. *graminearum* wild-type Z3643 strain; ΔFGSG_02052, a transgenic Z3643 strain lacking the FGSG_02052 gene. The perithecia formed 7, 8, 9, or 10 days after perithecia induction, and the asci and ascospores that developed within 8-day-old-perithecium were observed through a dissecting microscope (left panels) and differential interference contrast microscope (right panels), respectively. The cirrhi production from the perithecia was indicated by arrows. Scale bars = 100 μm for perithecia and 20 μm for asci/ascospores.

The targeted deletion of FGSG_00348 (designated *FgSMS-*2) from Z3643, which exhibited sequence similarity to a gene encoding an Argonaute protein known to participate in the RNA interference (RNAi) pathway in *Drosophila melanogaster* [31] and *N*. *crassa* [32], caused no dramatic changes in major traits such as hyphal growth, conidiation, pigmentation, virulence, and perithecia formation in *F*. *graminearum*. Unlike the perithecia produced in WT, those in Δ*FgSMS-*2 produced no cirrhi (ascospores oozing from the perithecia) at the ostiole 10 days after perithecial induction, and contained fewer numbers of asci that were formed at least 2 days later than WT (Fig 4), as previously reported in the Z3639 strain [33]. The germination rate of ascospores was not significantly different from WT. Interestingly, outcrossing the Δ*FgSMS-2* strain as a male to the Δ*MAT1-2* strain as a female produced incomplete tetrads that mainly carried four ascospores rather than eight, in which the *GFP* marker did not segregate equally (Fig 5). Furthermore, the outcross between the Δ*FgSMS-2* strain (female) and Δ*MAT1-2* strain (male) produced asci similar to those in the self-cross of Δ*FgSMS-2* strain (Fig 5). qPCR confirmed that *FgSMS-2* was specifically expressed at a later stage (7 days after perithecial induction) of sexual development, and was regulated transcriptionally by both *MAT* loci (S6 Fig). In addition, the expression of three DEGs (FGSG_02877 and belonging to UUN, and 05906 to DDN) was upregulated during sexual development in the Δ*FgSMS-2* strain compared to the WT strain, suggesting that *FgSMS-2* is involved in the degradation of the mRNAs of these DEGs (S6 Fig). The deletion of another Argonaute-like gene (FGSG_08752) in the *F*. *graminearum* genome, which was not differentially regulated by the *MAT* loci, had no effect on sexual development; even the double deletion of *FgSMS-2* and FGSG_08752 yielded an identical phenotype as the Δ*FgSMS-2* strain (Fig 4). Unlike Δ*FgSMS-2*, the strain lacking FGSG_02052 produced cirrhi at least 2 days earlier than WT, but it produced as many normal-looking ascospores as WT (Fig 6).

Among the 25 genes with sexual development-specific functions, ten (40%; FGSG_04480, 00404, 01366, 11826, 05151, 06966, 08890, 08891, 08892, 08893), including four *MAT* genes, encode transcription factors, two (FGSG_08320 and 09182) encode SM gene cluster members, and the others are involved in metabolism (FGSG_13708, FGSG_03673, FGSG_07578, FGSG_06059), chromatin silencing (FGSG_13162), cell adhesion (FGSG_03916), signaling (FGSG_05239), cytoskeleton dynamics (FGSG_01862), RNA inference (FGSG_00348), or unknown functions (FGSG_11962 and FGSG_02052) (Table 2). Interestingly, 21 (84%) of the 25 sexual-specific genes were downregulated in Δ*MAT1-1*;Δ*MAT1-2*. Only 1 gene (FGSG_05239) among the 31 DDN-type genes examined had a sexual-specific function (Table 2).

In addition, 12 genes (FGSG_00532, 02572, 06039, 06228, 07368, 07546 [*MYT2*], 07869, 08795, 09019, 09834, 09896 [*GzICL1*], 10825] were responsible for pleiotropic phenotypes, including defects in sexual development. Three genes, which were not essential for sexual development, were involved in hyphal growth (FGSG_04946) or virulence toward the host plant (FGSG_05906 [*FGL1*], FGSG_10396]) (Table 2).

### Developmental time course expression of DEGs in the WT strain on carrot agar

A total of 37 DEGs identified in the current study were analyzed in the WT strain grown on carrot agar using qPCR to determine the time course of the transcriptional profiles during both the vegetative and sexual stages. Most DEGs examined in the current study (33 of 38) were confirmed as sexual development-specific at the transcriptional level, because they were expressed at higher or lower (for FGSG_05906 only) levels in the WT Z3643 strain under perithecial induction conditions compared to vegetative conditions (S12 Table). Among these, four genes (FGSG_ 05246, FGSG_05847, FGSG_06549, FGSG_01763) could be assumed to be involved in the early stage of perithecium formation, because their expression peaked 3 days after perithecial induction. In contrast, the remaining 28 genes are probably specific to a later stage of sexual development (S12 Table). However, the functional assignment of these genes relies on only a correlation, and needs further confirmation. In our previous study [28], 42 genes that were identified as DEGs in the current study were confirmed to be sexual development-specific in the WT Z3639 strain. Among these, 50% (21 genes) and 28.6% were DDD- and DDN-type, respectively (S8 Table).

When the DEGs in the current study were searched against the transcriptome data obtained from the fungal PH-1 strain at 6 developmental stages during perithecium formation, data revealed that 1,152 DEGs were expressed at these time points [23] (S13 Table). In particular, the transcript accumulation of 72% (87/121) of the XXD-type genes (including DDD, DND, NDD, NND) peaked 96 h after perithecial induction, whereas the expression of 70% of the XXN-type (DDN, DNN, NDN) genes peaked at earlier time points (2, 24, 48, or 72 h; S13 Table). However, the upregulated genes were not enriched at a specific stage. In addition, 45% (54/120) of the genes that were downregulated in OM2 exhibited the highest transcript accumulation at 72 h (Table 3).

**Table 3:** DEGs overlapped with the transcriptomics data during sexual developmental processes, generated by Sikhakolli et al [23].

Expression category	Numbers of the DEGs whose gene expression levels reached the highest peaks in the developmental time-course of perithecium formation						total
	2h	24h	48h	72h	96h	144h	
DDD	5	5	2	7	72	6	97
NDD	1	0	1	2	6	0	10
NND	0	2	0	1	2	0	5
DDN	28	83	70	40	72	32	325
DNN	20	32	20	8	21	6	107
NDN	5	14	15	10	15	4	63
NUN	24	17	14	21	11	24	111
UNN	11	8	14	26	9	32	100
UUN	21	30	33	33	16	55	188
UUU	3	1	1	1	0	1	7
NNU	0	0	0	1	0	0	1
NUU	0	1	0	2	0	0	3
UNU	0	1	0	0	0	0	1
D in OM2	8	6	14	54	1	37	120
U in OM2	0	0	0	1	3	1	5
							1,152

Refer to S13 Table for details.

### Identifying a putative binding site for MAT1-2-1 protein using protein binding microarrays (PBM)

**Fig 7:**
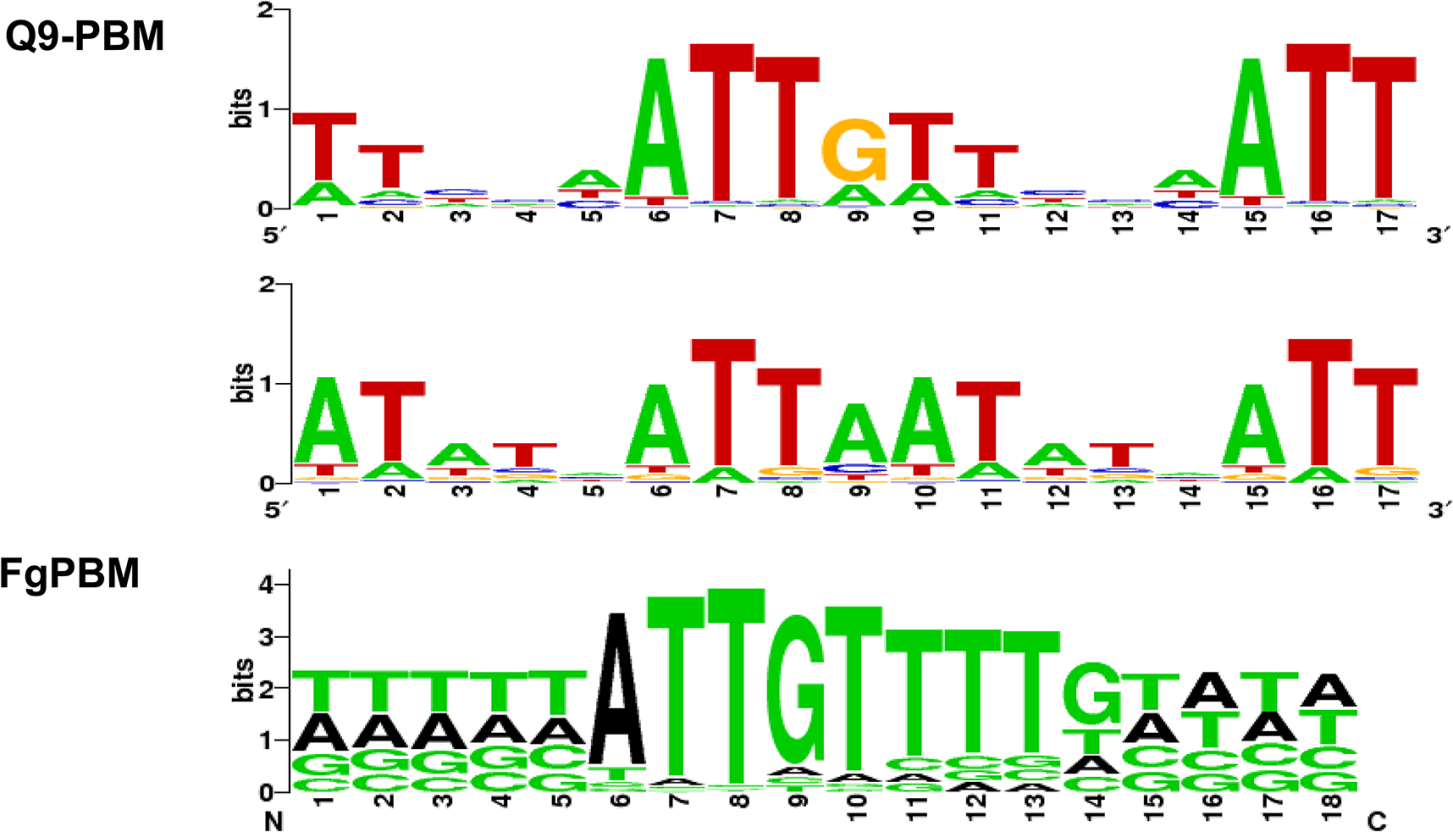
The determined consensus binding sequences according to two PBM analyses (Q9-PBM and FgPBM). The target probes in Q9-PBM (Quadruple 9-mer-Based PBM) are synthesized as quadruples of all possible 9-mer combinations, whereas FgPBM consists of probes designed from the putative promoter region (1,000 bp upstream of the start site of each ORF) of the *F*. *graminearum* genome (refer to S2 Text for details). To determine the binding motifs, two independent linear models were applied in the deep and heavy right tail region (refer to S2 Text and S7 Fig). Here, two of the most significant binding motifs identified by Q9-PBM are shown, among which the ATTGTT motif was also identified by FgPBM.

We used PBM technology [34, 35] to identify a putative binding site for the MAT1-2-1 protein (S7 and S8 Figs). Two PBMs (Q9-PBM, FgPBM) were hybridized to the DNA-binding HMG motif of MAT1-2-1, which was fused to DsRed fluorescent protein, and then expressed in *E*. *coli*. A comparison of the putative consensus binding sequences identified using both PBM methods indicated that ATTGTT could be the core binding sequence for the HMG domain of MAT1-2-1 (Fig 7), which is complementary to the core-binding element (AACAAT) of the mammalian sex-determining region Y (*SRY*) or *SRY*-related HMG box gene (*SOX*) [36–38].

Electrophoretic mobility shift assay (EMSA) revealed that the quadruple sequences of the identified motifs (ATTAAT and ATTGTT) had binding activities for the MAT1-2-1 HMG box domain (S9A Fig). The promoter regions of three genes (FGSG_04946, FGSG_08467 and FGSG_06480) could bind to MAT1-2-1, but their binding activities were relatively weak (S9B Fig). Experimental details and discussion from the PBM and EMSA analyses were described in S2 Text.

### Transcriptional networks of the genes regulated by the *MAT* loci

**Fig 8:**
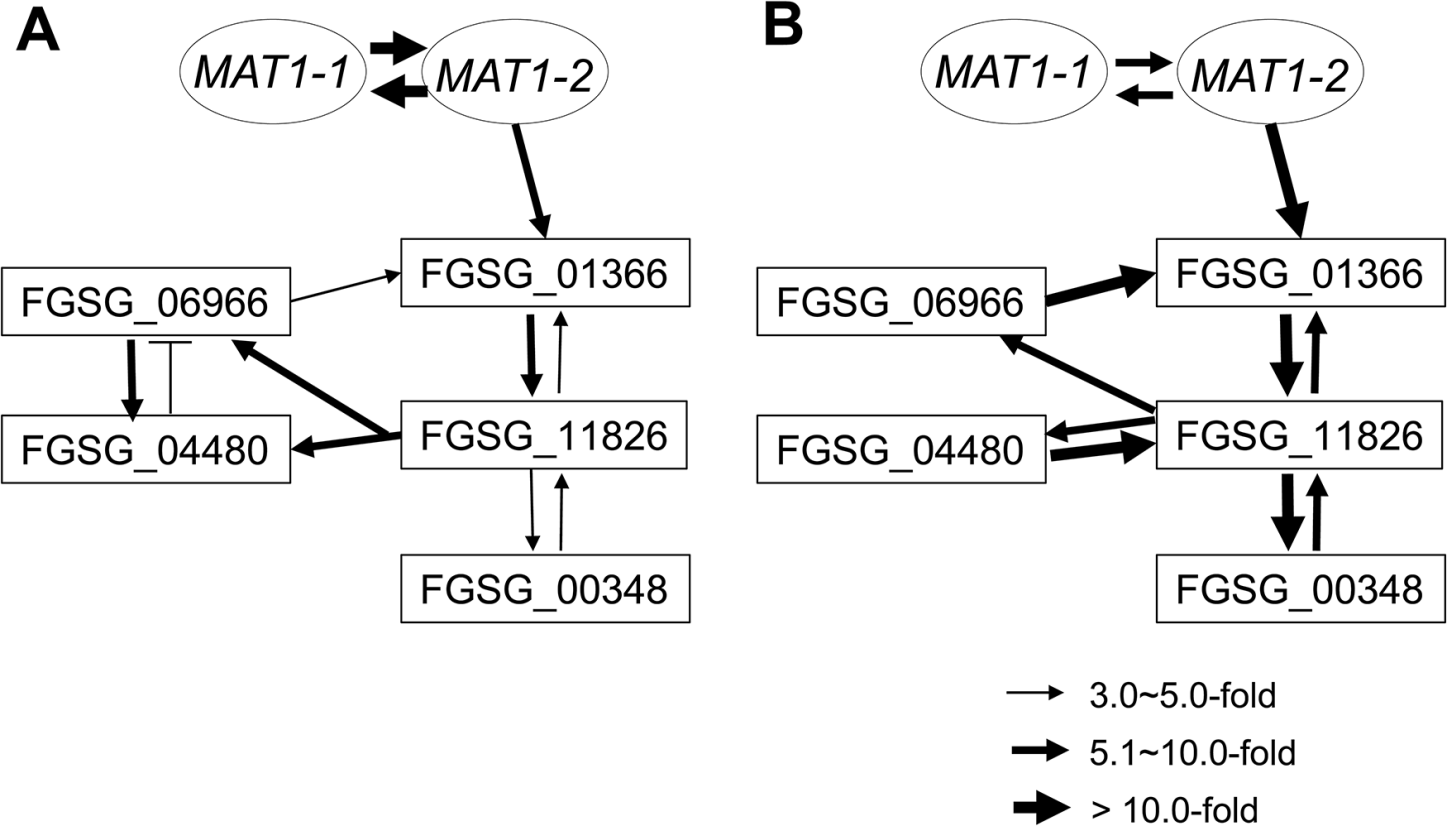
Regulatory networks among the sexual transcription factors (TFs) and an RNAi regulator (FGSG_00348) under control of the *MAT* loci during sexual development in *F*. *graminearum*. A map of the regulatory interactions among these genes was constructed based on the gene expression patterns in the fungal strains deleted for each regulator during sexual developmental processes [3- (A) and 6- (B) days after perithecial induction on carrot agar] (Table 4).

To assess the possible regulatory relationship among the sexual development-specific *MAT* downstream regulator genes (four transcription factors and one RNAi regulator), we used qPCR to examine their expression patterns in fungal strains in which each gene was deleted during sexual development (3 and 6 days after perithecial induction) (Table 4). Using a fold-change threshold of 3.0, because most genes were expressed at relatively low levels (based on previous transcriptome data [23]), a map of the regulatory interactions among the genes was constructed, as previously performed [39]. Because the core binding sequence of MAT1-2-1 was found in only FGSG_01366, the transcription factor carrying the HMG-box motif, we assumed that FGSG_01366 is the first putative target of MAT1-2-1 in the network (Fig 8). However, the deletion of FGSG_06966, and FGSG_11826 had a significant effect on the expression of FGSG_01366 and other regulatory genes, suggesting that interregulatory networks operate among these genes. Interestingly FGSG_00348, which encodes an Argonaute-like protein was downregulated in strains in which all four transcription factor genes had been individually deleted, suggesting that it was a downstream target of these genes. In addition, none of the target gene deletions had a significant effect on the transcription of *MAT1-1-1* and *MAT1-2-1*, which confirms that these regulatory genes are downstream of the *MAT* loci in *F*. *graminearum*.

**Table 4:** Expressiona of sexual-specific MAT-target regulator genes in the *F*. *graminearum* strains with the corresponding gene deletions.

	Strains with the gene deletions						
Gene	ΔFGSG_00348	ΔFGSG_01366	ΔFGSG_04480	ΔFGSG_06966	ΔFGSG_11826	Δ*MAT1-1*	Δ*MAT1-2*
**3 days after perithecial induction**							
FGSG_00348	–	↓(3.2)	NC(1.9)	↓(4.4)	↓(3.2)	↓(4.3)	↓(3.2)
FGSG_01366 (TF)	↓(3.6)	–	↑(8.5)	↓(3.4)	↓(5.5)	↓(4.2)	↓(8.5)
FGSG_04480 (TF)	NC(1.8)	↓(4.9)	–	↓(8.1)	↓(5.8)	↓(9.7)	↓(3.9)
FGSG_06966 (TF)	NC(1.2)	NC(1.5)	↑(3.2)	–	↓(6.1)	↓(4.0)	↓(10.1)
FGSG_11826 (TF)	↓(4.2)	↓(6.3)	NC(0.8)	↓(4.7)	–	↓(29.2)	↓(19.7)
*MAT1-1-1* (TF)	NC(0.7)	NC(1.6)	NC(2.4)	NC(1.4)	NC(1.9)	–	↓(24.4)
*MAT1-2-1*(TF)	NC(0.9)	NC(1.1)	NC(2.5)	NC(1.4)	NC(2.4)	↓(14.2)	–
**6 days after perithecial induction**							
FGSG_00348	–	↓(40.5)	↓(22.0)	↓(4.1)	↓(39.6)	↓(81.4)	↓(125.5)
FGSG_01366 (TF)	NC(1.5)	–	NC(1.2)	↓(12.7)	↓(9.5)	↓(11.0)	↓(26.4)
FGSG_04480 (TF)	NC(1.0)	↓(3.3)	–	NC(1.5)	↓(9.6)	↓(23.1)	↓(13.5)
FGSG_06966 (TF)	NC(1.4)	NC(1.6)	NC(1.0)	–	↓(7.3)	↓(6.8)	↓(11.6)
FGSG_11826 (TF)	↓(7.7)	↓(12.4)	↓(10.3)	↓(15.8)	–	↓(33.4)	↓(26.2)
*MAT1-1-1* (TF)	NC(2.8)	NC(1.1)	NC(2.5)	NC(1.5)	NC(2.0)	–	↓(8.9)
*MAT1-2-1*(TF)	NC(1.8)	NC(1.3)	NC(1.9)	NC(1.9)	NC(1.6)	↓(8.9)	–

^a^determined by qPCR using total RNAs from the fungal cultures grown on carrot agar 72 h after perithecial induction.

The number in parenthesis is the average fold-change (FC) with a *p*-value ≤ 0.05 in gene expression between the wild-type and a deletion strain. Arrows indicate down- (↓) or up-regulation (↑).

ΔFGSG#: the deletion mutant of each gene ID at the *F*. *graminearum* genome database, TF: transcription factor, NC: no changed determined by FC <3.0.

## Discussion

### Identification of DEGs under control of *MAT* loci

The most significant achievement in this study is that it provided a comprehensive investigation of the putative target genes of the *MAT* loci during sexual development in self-fertile *F*. *graminearum*. Genome-wide transcriptional profiling in various *MAT* genetic backgrounds, and subsequent in-depth and high-throughput analyses allowed us to explore the regulatory networks and function of *MAT-*target genes during the early sexual developmental process when the major regulators (*MAT1-1-1*, *MAT1-2-1*) of each *MAT* locus are expressed at their peak levels [14]. Similar to other filamentous ascomycetes, *F*. *graminearum* undergoes various cellular processes during each stage of sexual development including mating, cell fusion, nuclear division, fusion, meiosis, ascus/ascospore development, and perithecium maturation. These aspects of sexual development could include pheromone-mediated membrane function, signal transduction, cytoskeleton dynamics, secretory pathways, cell cycle, cell adhesion, apoptosis, and differentiation [40].

The GO terms associated with genes that are differentially expressed in strains carrying a single *MAT* gene or no *MAT* gene were significantly enriched for terms related to sexual development processes. In particular, the terms of metabolism including cell wall organization, developmental processes involved in reproduction, and the cellular response to chemical stimulus were enriched among the DDN- and UUN- type DEGs, which are the most frequent groups. Similarly, those described above as well as related to signaling, cellular homeostasis, and cell cycle were enriched among the DEGs that were found in only Δ*MAT1-1* or Δ*MAT1-2* (DNN-, UNN-, NDN-, and NUN-types).

Unlike the DEGs described above, those from the *MAT-*null strain (mostly DDD-type) were orthologous to the genes associated with the GO terms that were more specific to sexual development (S3 Table). These genes, most of which were also *MAT* loci-specific, play important roles in various stages of sexual development ranging from mating to fruiting body formation, which was further confirmed using high-throughput gene deletions. The high frequency of genes specific to sexual development (86.5%; 33 of 38 according to qPCR) among the DEGs suggests that most *MAT*-target genes are involved in sexual development. A comparison with the study by Hallen et al. [22] identified 169 additional DEGs that were specifically induced during sexual development, which further supports the transcriptional specificity of *MAT*-target genes.

### The *MAT* loci regulate the pheromone/receptor system

**Fig 9:**
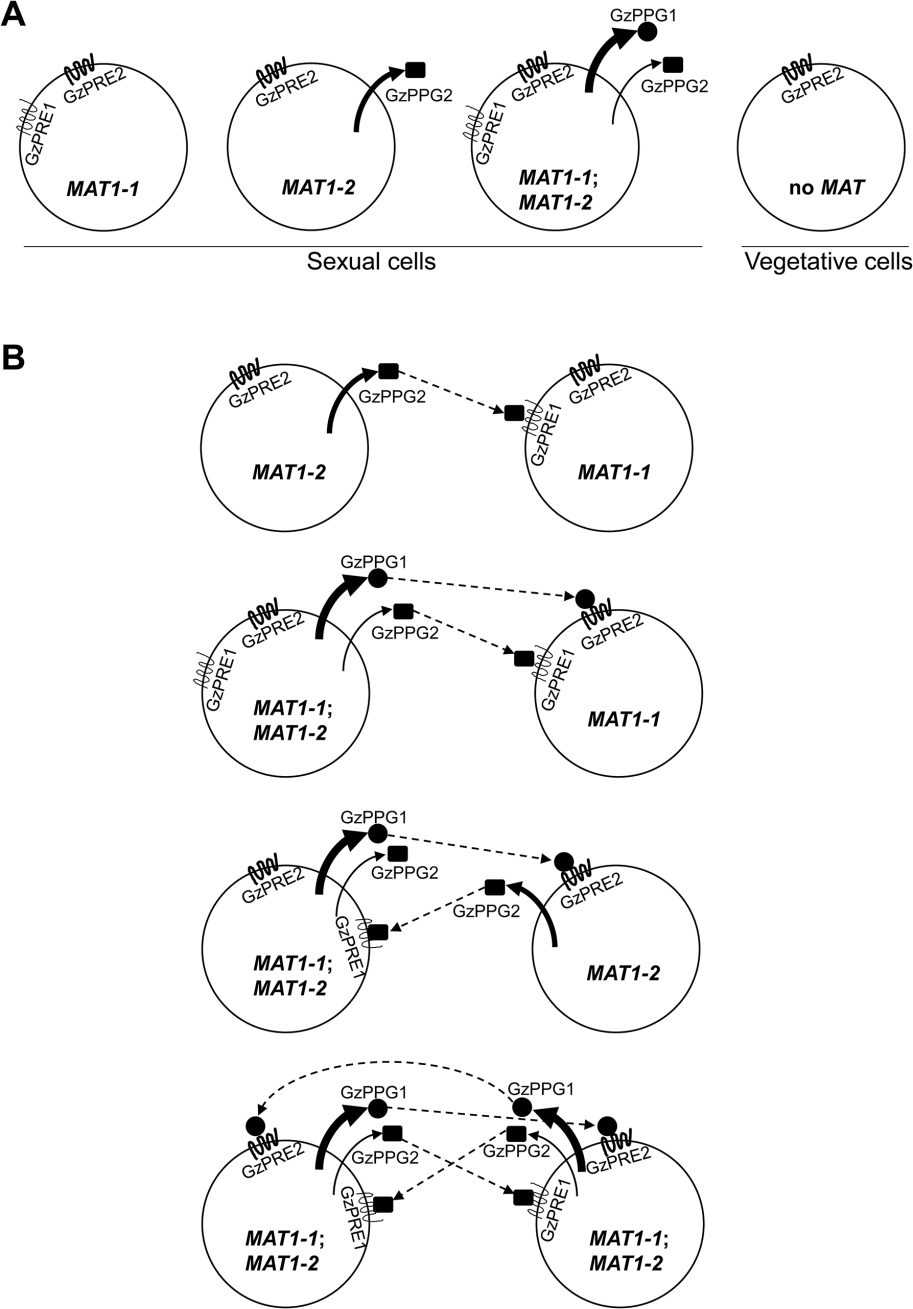
Diagrams for the expression of pheromone precursors and their cognate receptors in the *F*. *graminearum* Z3643 strain and its *MAT*-deletion strains used in this study (A), and for all of the possible interactions between the fungal cells or nuclei for mating in *F*. *graminearum* (B). These diagrams were constructed based on the differential *MAT*-locus expression in various *MAT*-deletion strains determined by qPCR (Table 1). The *MAT* genotypes in the *F*. *graminearum* strains are indicated in a circle depicting a fungal cell. *MAT1-1;MAT1-2*, a self-fertile *F*. *graminearum* wild-type (WT) strain carrying both *MAT1-1* and *MAT1-2* loci; *MAT1-1*, a self-sterile strain lacking the *MAT1-2* locus; *MAT1-2*, a self-sterile strain lacking *MAT1-1*; no *MAT*, a WT strain not expressing both *MAT* loci. Sexual cells, fungal cells grown under the sexual condition, where *MAT* gene expression is specifically induced. Vegetative cells: fungal cells during the vegetative growth stage, where no *MAT* expression occurred. The possible interactions between pheromone precursors and their cognate receptors are indicated by dashed arrows.

It is unclear if or how mating identity is maintained by the *MAT-*specific pheromone/receptor system in homothallic *F*. *graminearum*, although it was suggested that the single (opposite) *MAT*-mediated intercellular recognition might occur by differential expression of both *MAT* loci (S10 Fig) [41]. However, in the current study, qPCR of various *MAT*-deletion strains suggested *MAT1-2*-locus*-*specific *GzPPG2* expression and *MAT1-1*-locus*-*specific *GzPRE1* expression (Fig 9). In the homothallic *S*. *macrospora*, the pheromone genes *ppg1* and *ppg2* were downregulated in the ΔSmtA-1 (Δ*MAT1-1-1*) strain, whereas only *ppg2* was downregulated in the ΔSmta-1 strain (Δ*MAT1-2-1*) [15]. However, it is unknown if the expression pattern of *ppg* in *S*. *macrospora* is *MAT*-specific, because no studies have been performed in strains lacking the *MAT* loci. Despite the dispensability of pheromones and their receptors for the formation of fertile perithecia in *F*. *graminearum* [30], the qPCR results in the current study predict a role for this system during sexual developmental processes (e.g., pheromone-mediated fertilization or inter-nuclear recognition) in *F*. *graminearum* (Fig 9).

Once perithecia formation has been induced, WT cells, which carry and express both *MAT1-1;MAT1-2* [14], are capable of producing two pheromone precursors (GzPPG1, GzPPG2) and their cognate receptors (GzPRE1, GzPRE2). However, during the vegetative growth stage, WT cells carrying but not expressing both *MAT* loci [14], which are comparable to *MAT*-null cells during the sexual stage, produced no pheromones or receptors, except for GzPRE2. (Table 1, Fig 9A). Cells carrying only the *MAT1-1* locus (the Δ*MAT1-2* strain in this study), which could be present among WT cells as well by differential expression of *MAT* loci (S10 Fig), if occurs, produce only two pheromone receptors (GzPRE1 and GzPRE2). In contrast, those cells carrying only the *MAT1-2* locus can produce the pheromone GzPPG2 as well as the receptor GzPRE2 (Fig 9A). If the exclusive expression of one *MAT* locus occurs within the parental cells (e.g., the conidia as the male element and the ascogonium as the female element) [40], we could hypothesize that an interaction between two cells is required for fertilization: a cell with only *MAT1-1* (designated *MAT1-1*) with one with *MAT1-2* (*MAT1-2*), *MAT1-1* with a WT cell (*MAT1-1*;*MAT1-2*), and *MAT1-2* with *MAT1-1*;*MAT1-2* (Fig 9B). In the first case, which usually occurs in heterothallic species, the interaction between the GzPPG2 pheromone released from *MAT1-2* and its cognate receptor GzPRE1 in *MAT1-1* might lead to the fertilization. In the other two cases, the interactions between GzPPG1/GzPRE2 and GzPPG2/GzPRE1 would lead to fertilization. Since *GzPPG1* is expressed in the germinating conidia of *F*. *graminearum* [42], it is possible that the *MAT1-2* and WT strains could act as the male parent in the fertilization model (Fig 9B). The possible fertilization in a heterothallic manner, as proposed in the first combination (*MAT1-1* vs. *MAT1-2*), was previously demonstrated by outcrosses between the Δ*MAT1-1*;*MAT1-2* and *MAT1-1*;Δ*MAT1-2* strains of *F*. *graminearum*, which produced fertile perithecia. However, their numbers and fertility were much lower than in the WT strain [11]. A recent study showed that outcrossing Δ*MAT1-1*;*MAT1-2* as the male parent with *MAT1-1*;Δ*MAT1-2* as the female produced partially normal perithecia, whereas an outcross with the reverse parental roles (i.e., Δ*MAT1-1*;*MAT1-2* female × *MAT1-1*;Δ*MAT1-2* male) produced only small, sterile perithecia [13]. The reduced fertility of perithecia in these outcrosses could be attributed to the low level of expression of *GzPRE1* in *MAT1-1* strain. The failure in the latter outcross could be ascribed to the lack of (or poor) ability of the *MAT1-1* strain to produce pheromones as the male parent. The other two combinations for fertilization (Fig 9B) could also yield a successful outcross between WT (male) and either the Δ*MAT1-1*;*MAT1-2* or *MAT1-1*;Δ*MAT1-2* strain (female), as described previously [11]. The retained sexual ability of the *F*. *graminearum* strain lacking all of the pheromone and receptor genes suggests that *F*. *graminearum* might mate randomly. In addition, the current results reveal the possibility that pheromone-mediated fertilization and/or internucleus recognition occur after random mating in *F*. *graminearum*.

The similar effect of Δ*MAT1-2* and Δ*MAT1-1;*Δ*MAT1-2* on the expression of two pheromone genes (*i*.*e*., downregulation of both *GzPPG1* and *GzPPG2*) could be attributed to the downregulation of *MAT1-1* genes in the Δ*MAT1-2* strain. However, the effect of Δ*MAT1-1* on the expression of *GzPPG2* (*i*.*e*., upregulation) was opposite that of Δ*MAT1-2* or Δ*MAT1-1;*Δ*MAT1-2*, suggesting that the effect of Δ*MAT1-1* on the expression of *MAT1-2* might not be as great as the effect of Δ*MAT1-2* on *MAT1-1* expression.

### Large-scale functional analysis of the selected DEGs

The phenotypic characterization of transgenic strains lacking the selected DEGs provides clear evidence for their functional requirement and specificity for sexual development, as well as the potential stage of their roles during sexual development. Among the 25 genes that were specific to sexual development, 14 (56%) affected the perithecium initial or protoperithecium formation, because the gene deletion strains produced barren protoperithecium (that did not develop further into fertile perithecium), or no perithecium initials (Table 2). This strongly suggests that the *MAT* loci regulate the genes that are necessary for the cellular events involved in the developmental stages that occur before nuclear fusion and/or meiosis that may subsequently facilitate the formation of mature perithecium and asci/ascospores (S10 Fig). Based on sequence homology, the cellular functions of these sexual-specific genes could be proposed as follows. Two G-protein coupled receptors (the pheromone receptor GzPRE2 and FGSG_05239) might be involved in mating and subsequent signal transduction. Ten TFs, including four *MAT* genes, might regulate many downstream genes that are necessary for protoperithecium formation. An additional two TFs (FGSG_06228, FGSG_03597), which encode a regulator of G-protein signaling (RGS) protein domain, were upregulated by *MAT* deletions (UNN- and UUN-type, respectively). Both genes had relatively high sequence similarity to the RGS gene in *Aspergillus nidulans*, designated *FlbA* (with 61% and 43% amino acid identities, respectively), which is required for the control of mycelial proliferation and asexual sporulation [43]. FGSG_03597 is also downregulated in OM2. These expression patterns suggest that the *MAT1-1* locus might suppress the RGS genes during early sexual development to block the signal transduction pathways required for asexual sporulation.

The suppression of asexual sporulation throughout sexual development has been demonstrated in *F*. *graminearum*; conidiation in the self-sterile Δ*FgVelB* strain was derepressed even during perithecial formation [28]. It is likely that at least one (FGSG_06228) of these genes is the functional homolog of *A*. *nidulans FlbA* because the targeted deletion of FGSG_06228 had a significant effect (*i*.*e*. an ~11-fold reduction) on conidiation in *F*. *graminearum* Z3643, which was different from the phenotypic changes in the Z3639 genetic background [24]. In addition, ΔFGSG_06228 exhibited pleiotropic changes in other traits such as perithecium formation, virulence, and mycotoxin production (S11 Table). Importantly, this study is the first to show inactivation of the *FlbA*-like gene by the *MAT* loci during sexual development in filamentous ascomycetes, although the regulatory networks for conidiation in which *FlbA* plays an important role have been studied in *Aspergillus* [44]. Since the *MAT* genes were downregulated in the Δ*FgVelB* strain, it is possible that FgVelB, a component of the FgVeA complex, controls asexual sporulation by activating the *MAT* loci. Consistent with this notion, *FgVelB* controls the expression of *MAT* genes in *F*. *graminearum* [28].

FGSG_07368, a TF that represses ZEA biosynthesis [24], is involved in both ZEA production and perithecium formation because its deletion strain produced ~three-fold higher amounts of ZEA, but formed lower numbers of fertile perithecia compared to WT. This gene is downregulated in the OM2 strain, suggesting that the MAT1-2-1 protein represses ZEA biosynthesis during the perithecia induction stage by suppressing the expression of FGSG_07368. Because the negative effect of ZEA overproduction on sexual development was confirmed in *F*. *graminearum* [45], this *MAT-*induced regulation pattern suggests that the *MAT* loci acts as a master regulator to both activate and repress the expression of many genes during sexual development.

Seven genes (FGSG_08320, 13708, 06059, 07578, 07869, 03673, 09182) encode enzymes that might participate in the metabolic pathways that are required for protoperithecium formation. In particular, FGSG_09182 (*PKS3*) and FGSG_08320 are required for perithecial pigment [46] and an unidentified secondary metabolite, respectively. Four genes (FGSG_03916, 10742, 05246, 01862) might be involved in various cellular events such as cell adhesion (FGSG_03916), cellular fusion (FGSG_10742), paraphyses senescence (FGSG_05246), and cytoskeletal organization (FGSG_01862). FGSG_13162, which encodes a protein that controls the histone promoter, might be involved in chromatin silencing and could be required for the regulation of gene expression during protoperithecium formation. Sexual-specific genes are more required for the early stage of sexual development (e.g., the initial perithecium formation) than for postfertilization or post-meiotic events. This suggests that the *MAT* genes, particularly *MAT1-1-1* and *MAT1-2-1*, whose transcripts accumulate at the highest levels during the early stage [13, 14], control the genes that are involved in this stage more than those that regulate the later stages (e.g., nuclear fusion, meiosis, ascus maturation). However, it could also be attributed to the time point (48 h after perithecium induction) at which total RNA samples were extracted for microarray analysis. Nevertheless, the possibility that the *MAT* loci regulate both the early and late stages of sexual development is supported by the phenotypes of transgenic strains that lack each of three remaining genes (FGSG_00348, 00532, 02052). Specifically, these strains could produce mature perithecium, although the number of perithecia produced was lower (FGSG_00348) or higher (FGSG_00532) than WT. This suggests that the *MAT* genes also control the expression of the genes that are required for post-fertilization processes.

In particular, the deduced product of FGSG_00348, designated FgSMS-2, contains the PAZ- and Piwi domains that are also found in SMS-2 from *N*. *crassa* and AGO2 (Argonaute) from *D*. *melanogaster*. These proteins play an important role in the RNA silencing known as meiotic silencing by unpaired DNA (MSUD) and siRNA-directed RNA interference, respectively [32, 47]; they are the active part of the RNA-induced silencing complex and required for cleaving the target mRNA. The formation of asci carrying an incomplete set of ascospores or abnormally shaped ascospores in the Δ*FgSMS-2* strain suggests that the *MAT* loci regulate the meiotic events that lead to asci/ascospore development by activating the expression of *FgSMS-2*, which might control the RNAi pathway in *F*. *graminearum*. Another *SMS-2* ortholog (FGSG_0872) is present in the *F*. *graminearum* genome, which is similar to the *N*. *crassa* gene *Qde-2*, which regulates another RNA silencing pathway (quelling) during vegetative growth. However, the lack of transcriptional control of FGSG_0872 by *MAT* and the phenotypic changes caused by gene deletion suggest that *FgSMS-2* is the only Argonaute-like gene that controls meiosis and the subsequent developmental pathways in *F*. *graminearum*. The upregulation of three DEGs caused by Δ*FgSMS-2* during perithecial formation (S6 Fig) suggests that FgSMS-2 is involved in the degradation of the mRNA of these genes in *F*. *graminearum*, which is comparable to the role of Argonaute proteins. However, further investigations are necessary for confirmation, such as exploring and characterizing small RNAs.

Interestingly, 17 of the 21 sexual-specific genes (excluding 4 *MAT* genes) were downregulated in the *MAT*-null strain as well as Δ*MAT1-1* and Δ*MAT1-2*. The four remaining genes included FGSG_03916, which is repressed during sexual development, and FGSG_02655 (*GzPRE2*) and FGSG_00404, which are both differentially regulated only in OM2. These data suggest that all of the genes with sexual-specific functions are regulated by both the *MAT1-1* and *MAT1-2* loci. This also indicates that the presence of both MAT1-1 and MAT1-2 proteins within a separate or common nucleus is necessary for controlling most stages of sexual development, from ascogonium formation to perithecium maturation (S10 Fig). In addition, the high frequency (72%) of enrichment of XXD-type DEGs at a later stage (96 h after perithecial induction), which is dramatically different from the XXN-type DEGs enriched at the earlier stages (Table 3), strongly suggests that both *MAT1-1* and *MAT1-2* are required to control stages of development later than fertilization. However, the functional assignment of these DEGs into particular growth stages is based on only developmental time course gene expression, and awaits further confirmation.

### The regulation of sexual development by *MAT* in *F*. *graminearum*

**Fig 10:**
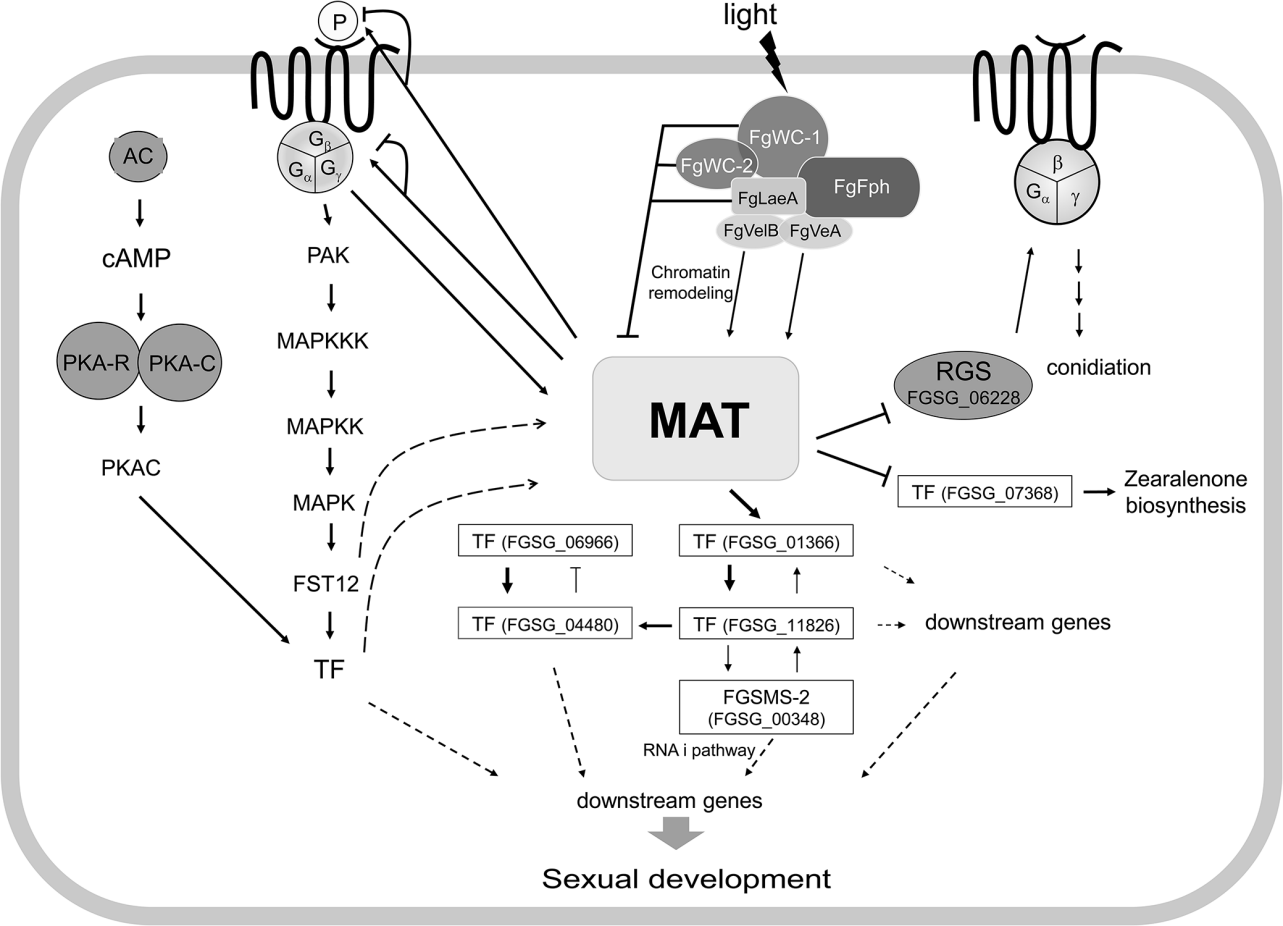
Schematic diagram of possible regulatory pathways of *MAT*-mediated sexual development in *F*. *graminearum*. The expression of both *MAT* loci are regulated by several environmental cues (e.g., light), probably via chromatin remodeling by the FgVeA complex and/or G-protein signaling pathways, and control the expression of putative target genes via regulatory cascades and/or networks involving several downstream transcription factors (TFs) and an RNAi regulator (FgSMS-2). Abbreviations: MAT, mating-type proteins encoded by both *MAT1-1* and *MAT1-2* loci; AC, adenyl cyclase; PKA-R, protein kinase A regulatory subunit; PKA-C, protein kinase A catalytic subunit; PAK, protein kinase; FST12, an *F*. *graminearum* ortholgoue of the yeast STE12; RGS, Regulator of G-protein signaling; P, pheromones. Solid lines with triangular and flattened arrowheads represent gene activation and repression, respectively. The dashed arrows indicate possible regulatory directions, not determined by qPCR.

Taken together, the results of the current and previous studies provide insights toward a comprehensive understanding of the *MAT-*mediated regulatory pathways that control sexual development in *F*. *graminearum* (Fig 10). The environmental cues for sexual development elucidated to date in *F*. *graminearum* are light, nutrient conditions (probably nitrogen starvation), and mycelial autophagy [40]. In contrast to *A*. *nidulans*, *F*. *graminearum* only produces fertile perithecia on carrot agar in the presence of light. The molecular characterization of *F*. *graminearum* orthologs (*FgWC-1* [FGSG_07941] and *FgWC-2* [FGSG_00710]) of the photoreceptors encoding *wc-1* and *wc-2* in *N*. *crassa* revealed that these two genes repress the expression of *MAT* genes during the sexual development (within 3−5 days of perithecial induction) [48]. Similarly, *FgLaeA*, a component of the FgVeA complex that is involved in chromatin remodeling, is also likely to repress *MAT* gene expression, because the *MAT* genes were upregulated in the Δ*FgLaeA* strain [49]. However, other members of the FgVeA complex, FgVeA and FgVelB, activate *MAT* gene expression because the *MAT* genes were downregulated in the Δ*FgVelB* strain, and the Δ*FgVeA* strain was self-sterile [28]. Taken together, it is possible to speculate that the expression of the *MAT* genes could be activated or repressed by chromatin remodeling via FgWC-1/FgWC-2 and FgVeA complexes. However, this speculation awaits further investigation. The downregulation of *MAT* genes in the Δ*GzGPA1* strain [33] suggests that they can also be positively regulated by a G-protein-mediated signal transduction pathway, which is probably induced via nutrient sensing. The deletion of most of the genes in the G-protein signaling pathway, including the MAP kinase and cAMP cascades, caused dramatic changes in several traits, including sexual development [50–54]. This suggests that the *MAT* loci might be under the control of these signaling pathways. In contrast, most of the genes involved in these signaling pathways (S14 Table) were not differentially regulated in the *MAT-*deletion strains examined in this study. This suggests that *MAT* does not regulate these pathways.

Once activated under perithecial induction conditions [14], *MAT* genes control the expression of the pheromone/receptor system in a *MAT*-specific manner (particularly for *GzPPG2* and its cognate receptor *GzPRE1*), suggesting that *MAT* plays a role in pheromone-mediated fertilization. However, it is unclear if the maintenance of cell identity for mating, which might be mediated by the *MAT*-specific pheromone/receptor system and/or by a single *MAT* locus, occurs in *F*. *graminearum*, although the differential expression of each *MAT* locus within a single nucleus has been suggested in this regard. It is still possible that the pheromone/receptor system plays an important role in the stages after fertilization, such as during inter-nuclear recognition.

Based on the genome and promoter microarray results in the current study, it is possible that the regulation of sexual development under the control of the *MAT* loci might involve cascades or networks that are downstream of TFs. Focusing on the TFs that are specific to sexual development at both the gene expression and functional levels allows a model of regulation of these TFs by the *MAT* loci to be proposed (Fig 10). Although inter-regulatory networks between these TFs have also been proposed, it is possible that these major TFs control the expression of the genes that are involved in various molecular processes related to sexual development, conidiation, and zearalenone production. In this regulatory circuit, the HMG-box motif containing TF FGSG_01366 is the main downstream regulator of the *MAT* loci. Interestingly, FGSG_01366 is an ortholog of *PaHMG5* in *P*. *anserina*, which plays a crucial role in a network containing several HMG-box factors that regulate mating-type genes and their target genes [39]. However, the regulatory roles of these HMG-box TFs for sexual development differ between the two species. FGSG_01366 is a downstream target of the *MAT* loci in *F*. *graminearum*, whereas *PaHMG5* is an upstream regulator of mating-type genes (*FMR1* and *FPR1*, which are comparable to *MAT1-1-1* and *MAT1-2-1*, respectively) and the pheromone/receptor system [39]. The expression of the *F*. *graminearum* ortholog (FGSG_05151) of the other HMG box gene (*PaHMG8*), a downstream target of *PaHMG5*, in *P*. *anserina* was also downregulated in the *MAT-*null background (NND-type). This suggests that FGSG_05151 is a member of the regulatory circuit of TFs that are under the control of the *MAT* loci (Fig 10), although the gene deletion phenotype was not as dramatic as that for FGSG_01366 (Table 2). The other regulatory mechanism under the control of the *MAT* loci might be the RNAi pathway involved in meiosis and/or ascus development. Although RNAi-mediated MSUD was not demonstrated in *F*. *graminearum*, the functional requirement of the Argonaute-like protein FgSMS-2 for ascus maturation and ascospore morphology and upregulation of several DEGs by Δ*FgSMS-2* suggests that RNAi may have a regulatory role during meiosis in *F*. *graminearum*.

The time course expression pattern and deletion of selected DEGs suggest that major *MAT* genes (*MAT1-1-1*, *MAT1-2-1*), which are highly induced at an early stage, regulate the genes involved in the later as well as early stages of sexual development (e.g., meiosis, ascospore formation, discharge). This suggests that *MAT* loci play crucial roles throughout sexual development in *F*. *graminearum*.

In summary, both *MAT* loci are activated by several environmental cues via chromatin remodeling and/or signaling pathways, and then control the expression of at least 1,245 target genes during the early stage of sexual development via regulatory cascades and/or networks involving several downstream TFs and RNAi. The regulatory effects of the *MAT* loci on these target genes could be directly achieved by the binding of MAT1-2-1 protein to core sequences within the promoter regions of some target genes, or indirectly via downstream TFs.

## Materials and Methods

### Fungal strains and culture conditions

The *F*. *graminearum* WT strains PH-1 and Z3643 [55], which belong to lineage seven of the *F*. *graminearum* species complex [5, 17], are self-fertile. T43ΔM1-3 [25], T43ΔM2-2 [17], and T43ΔDM1M2 (S1 Fig) are self-sterile transgenic mutants derived from Z3643, and lack *MAT1-1*, *MAT1-2*, and both *MAT1-1* and *MAT1-2* loci, respectively. The Δ*MAT1-1-1*, Δ*MAT1-1-2*, and Δ*MAT1-1-3* strains were previously generated by deleting the *MAT1-1-1*, *MAT1-1-2*, and *MAT1-1-3* transcripts at the *MAT1-1* locus of Z3643, respectively; they are all self-sterile [14]. OM2 (S2 Fig) is a *MAT1-2-1*-overexpressing strain that was generated by insertion of *MAT1-2-1* into the T43ΔM2-2 strain under the control of the *F*. *fujikuroi EF1A* promoter. The WT and transgenic strains were stored in 20% glycerol at –70°C. Sexual development was induced on carrot agar, as previously described [11, 56]. For genomic DNA extraction, each strain was grown in 50 mL CM [56] at 25°C for 72 h on a rotary shaker (150 rpm). Conidiation was induced in CMC liquid medium [57].

### Nucleic acid manipulations, primers, and qPCR

Fungal genomic DNA was prepared as previously described [56, 58], and total RNA was extracted from mycelia and/or protoperithecia that had formed on carrot agar using an Easy-Spin Total RNA Extraction kit (Intron Biotech, Seongnam, Korea) according to the manufacturer’s instructions. All of the PCR primers used in this study (S15 Table) were synthesized by Bioneer Corporation (Chungwon, Korea). DNA gel blots were prepared [59] and hybridized using biotinylated DNA probes that were prepared using the BioPrime DNA labeling system (Invitrogen, Carlsbad, CA, USA), and were developed using the BrightStar BioDetect Kit (Ambion, Austin, TX, USA). Other general procedures for nucleic acid manipulations were performed as previously described [59]. qPCR was performed using SYBR Green Super Mix (Bio-Rad) with first-strand cDNA synthesized from total RNA [33, 60]. The amplification efficiency of all genes was determined as previously described [60]. Gene expression was measured in three biological replicates from each time point. *EF1A* (FGSG_08811) was used as an endogenous control for data normalization [60].

### Luciferase assays and virulence tests

Fungal strains were grown in 20 mL agmatine-amended liquid medium [61] for trichothecene production and SG liquid medium [62] for zearalenone production, as previously described. Luciferase activity was then measured in cell lysates from the strains using GloMax 96 Microplate Luminometer (Promega) as previously described [63]. The virulence of the fungal strains was determined on wheat heads as previously described [49].

### Microarrays

Total RNAs were isolated from carrot agar cultures of the *F*. *graminearum* Z3643 WT strain carrying both *MAT1-1* and *MAT1-2*, three *MAT-*deletion strains (Δ*MAT1-1* [carrying only *MAT1-2*], Δ*MAT1-2* [carrying only *MAT1-1*], and Δ*MAT1-1*;ΔMAT1-2), and the OM2 strain, which had been grown for 2 days after sexual induction. Double strand cDNAs were synthesized as previously described [33]. Microarray analysis was conducted at GreenGene Biotech (Yongin, Korea) using the *F*. *graminearum* microarray that was manufactured at NimbleGen (Madison, WI, USA), as previously described [33]. This array includes 13,382 transcripts from *F*. *graminearum* sequencing assembly three. Experiments were repeated three times with total RNA samples that were independently prepared. Microarrays were scanned using Genepix 4000 B (Axon Instruments, Toronto, Canada), and the signals were analyzed with Nimblescan 2.5 (NimbleGen).

The data were normalized and processed with cubic spline normalization using quantiles to adjust for signal variation among chips [64]. Probe-level summarization using Robust Multi-Chip Analysis (RMA) with a median polish algorithm implemented in NimbleScan was used to produce call files. Multiple analyses were performed using the limma package in an R computing environment [65]. Genes with *P* ≤ 0.05 (significant) were collected and selected further for genes with expression > 1 or < −1 in at least one *MAT* genetic background compared to another background such as WT (*MAT1-1;MAT1-2*), or *MAT*-null (Δ*MAT1-1*;Δ*MAT1-2*). The entire dataset from the microarrays was deposited in the NCBI Gene Expression Omnibus (GEO) database (http://www.ncbi.nlm.nih.gov/geo) under the accession number GSE58543.

### GO analysis

The DEGs identified in this study were examined for significant enrichment of functional categorization using GO analysis [66]. The GO term enrichments were calculated with GOMINER [66, 67] (http://www.geneontology.org/, http://discover.nci.nih.gov/gominer/). The 11,603 genes were matched to *A*. *nidulans* FGSC A4 sequencing assembly (*Aspergillus* Genome Database; http://www.aspergillusgenome.org/) with score 30 and up by BlastP analysis, and were used as total gene set in gominer for GOMINER analysis. Gominer first categorize each gene according to their GO terms and mode of gene expressions either down- or up- regulation. It calculates p-values with the one-sided Fisher exact test for the number of categorized GO terms in the total. The GO terms with p-value less than 0.05 were considered significantly enriched among the DEGs, and used in further analysis.

### Targeted deletions of the selected DEGs

DNA constructs to delete individual DEGs from the genomes of *F*. *graminearum* Z3643 or PH-1 were created using a split marker recombination procedure, as previously described [68]. Generally, the 5′ and 3′ flanking regions of the target gene, which were amplified by PCR using the primers listed in S15 Table, were mixed with the *gen* or *hygB* cassettes, which were amplified from pII99 and pBCATPH using the primer pairs Gen-for/Gen-rev and Hyg-for/Hyg-rev, respectively. The final split markers were amplified from the mixture using the new nested primer sets (S15 Table). The amplified products were added into WT *F*. *graminearum* protoplasts for transformation, as previously described [14, 49]. Gene deletion was confirmed using either DNA gel blot hybridization or PCR (S11 Fig).

## Supporting Information

S1 Text Targeted deletion and overexpression of *MAT* genes. (DOCX)

S2 Text Identification of a putative binding site for MAT1-2-1 protein using protein binding microarrays (PBM) and Electrophoretic mobility shift assay (EMSA). (DOCX)

S1 Fig Targeted deletion of the entire *MAT* loci from the genome of the *F*. *graminearum* Z3643 strain. Left panel: deletion scheme. Z3643 (WT), genomic DNA of the *F*. *graminearum* wild-type Z3643 strain; T43ΔM1M2 (*MAT-*null), genomic DNA of a transgenic Z3643 strain lacking the entire *MAT* loci. Right: *Bgl*II-digested genomic DNA gel blot hybridized with the probe indicated in the deletion scheme. Lanes 1 and 2, Z3643 (WT); 3, T43ΔM1M2. DNA size markers are indicated on the right side of the gel.
(TIF)

S2 Fig Generation of a *MAT1-2-*overexpressing strain (OM2) of *F*. *graminearum*. (A) The scheme for insertion of the pOM2 vector, which carries a *MAT1-2-1* overexpressing construct, into the genome of a *F*. *graminearum MAT1-2-1-*deleted strain by a non-homologous gene integration event. Note that there is no homology between the pOM2 vector and Δ*MAT1-2-1* genome. The geneticin resistance gene (*gen*) in pII99 vector was used as a selectable marker in a co-transformation strategy. T43ΔM2–2 (Δ*MAT1-2-1*), genomic DNA of a transgenic *F*. *graminearum* Z3643 strain where the *MAT1-2-1* gene was replaced with the hygromycin B resistance gene (*hygB*); OM2, genomic DNA of a geneticin-resistant *F*. *graminearum* transformant carrying the *MAT1-2-1* overexpressing construct. P_*TEF1*_: *F*. *fujikuroi EF1A* promoter region. (B) *Hin*dIII-digested DNA gel blot of the OM2 strains, hybridized with a probe amplified from P_*TEF1*_. Lanes 1 and 2, OM2 strains showing different-, but larger-sized hybridizing bands than 4.8 kb (pOM2) (due to ectopic vector insertions). (C) Amplification of *MAT1-2-1* from total RNAs of the fungal strains by reverse transcription (RT) PCR. Lanes 1 and 2: RNA extracted from the *F*. *graminearum* wild-type Z3643 strain grown on carrot agar for 3 and 6 days, respectively (vegetative growth stage); lanes 3–4, those from the Δ*MAT1-2-1* strain grown on carrot agar for 3 and 6 days after removal of aerial mycelia, respectively (perithecial induction stage); lanes 5–6, those from OM2 [from lane 2 in (B)] under vegetative growth conditions; lanes 7–8, those from OM2 under the perithecia induction stage.
(TIF)

S3 Fig Perithecia formation in the Z3643 (WT), *MAT*-null, and OM2 strain on carrot agar. Vegetative growth, grown on carrot agar for 6 days; perithecia formation, grown on carrot agar 6 days after removal of aerial mycelia that had previously been grown for 6 days for vegetative growth.
(TIF)

S4 Fig Number of genes expressed differentially in the *F*. *graminearum* strains deleted for the *MAT1-1* locus (Δ*M1*;*M2*), *MAT1-2* locus (*M1*; Δ*M2*), and both *MAT1-1* and *MAT1-2* loci (Δ*M1*; Δ*M2*) compared to the *MAT-*null strain (Δ*M1*; Δ*M2*). DOWN, down-regulated genes; UP, up-regulated genes. The numbers of differentially regulated genes in each expression category are indicated above the category designations in parentheses (e.g., “DDN” means downregulation in Δ*MAT1-1*, downregulation in Δ*MAT1-2*, and no change in Δ*MAT1-1*;Δ*MAT1-2*).
(TIF)

S5 Fig Perithecium formation in the *F*. *graminearum* strains deleted for the selected DEGs, which led to changes in size or number of perithecia. (A) Perithecia of the *F*. *graminearum* strains on carrot agar plates 6 days after perithecial induction. WT, a wild-type Z3643 strain; ΔFGSG_XXXX, a transgenic Z3643 strain deleted for the gene with the corresponding gene ID (FGSG). Scale bar = 100 μm. (B) Average diameters of the perithecia produced by the *F*. *graminearum* strains shown in A, which were calculated from the measurement of 100 perithecia for each strain under a dissecting microscope. (C) Average number of perithecia/mm^2^ on a carrot agar plate. The different letter above bars represent significant differences (*p*<0.05) based on Tukey’s test.
(TIF)

S6 Fig Relative amounts of transcript accumulation of *MAT1-1-1*, *MAT1-2-1*, and *FgSMS-2* in WT (Z3643) (A) and *FgSMS-2* in *MAT-*deletion strains (B) grown on carrot agar for 6 days (vegetative growth), followed by perithecial induction for an additional 7 days (sexual growth). Relative amounts of transcript accumulation of three DEGs identified in this study in the Δ*FgSMS-2* strain grown on carrot agar (C), as described above. Δ*MAT1*, a transgenic Z3643 strain lacking the *MAT1-1* locus; Δ*MAT2*, a transgenic Z3643 strain lacking the *MAT1-2* locus; Δ*MAT1*Δ*MAT2*, a transgenic Z3643 strain lacking both *MAT* loci. Gene expression was measured in three biological replicates from each time point. *EF1A* (FGSG_08811) was used as an endogenous control for data normalization [60]. The amounts of *MAT1-1-1* (A) and *FgSMS-2* (B) transcripts from a 3-day-old vegetative sample of wild-type were used as a reference for comparison. Similarly, the amount of each gene transcript (C) from a 13-day-old sexual growth sample of WT was used as a reference for comparison. Note that the bars for transcript levels of FGSG_02877 and FGSG_02878 in the Δ*FgSMS-2* strain are broken because they are relatively larger than those in WT (C).
(TIF)

S7 Fig The rank-ordered signal distribution of the MAT1-2 PBM. Two independent linear models, y = ax+b, were applied in the deep (b1 = 26656.3, slope = -28.3) and heavy right (b1 = 1.154e+03, slope = - -9.719e-03) tail regions. Rank extrapolated is 2076.
(TIF)

S8 Fig Position weight matrix of the ATTAAT (A) or ATTGTTTAA (B) consensus motif. *P*-value for the motif enrichment is zero.
(TIF)

S9 Fig EMSA with ATTAAT (A) or ATTGTT (B) motif. The duplicated or quadruple 9 bp sequences containing these motifs (A), or 20 bp sequences of the putative promoter regions containing the motifs from the selected 16 genes (designated with FGSG_ ID) (B) were used for the interaction. EBNA: the Epstein-Barr nuclear antigen control system, cMAT1-2: HMG motif of the *F*. *graminearum* MAT1-2-1 protein expressed in *E*. *coli*.
(TIF)

S10 Fig Regulation of the DEGs identified in this study under control of the *MAT* loci during sexual developmental stages. The single *MAT* genotype (either *MAT1-1* or *MAT1-2*) of a fungal nucleus, depicted by a circle, is assumed based on the differential expression of both *MAT* loci. These nuclei may play a role in either intercellular or internuclei recognition. Both *MAT* loci, which could be expressed in both haploid and diploid cells, may control DEGs involved in meiosis and perithecia maturation. Abbreviations for differentially expressed genes with three characters: U, upregulated, D; downregulated; N, no change; X, either U or D. The first, second, and third characters represent the expression pattern in the Δ*MAT1-1*, Δ*MAT1-2*, and Δ*MAT1-1*;Δ*MAT1-2* strains, respectively, compared to the Z3643 strain. Solid lines with triangular and flattened arrowheads represent gene activation and repression, respectively.
(TIF)

S11 Fig Confirmation of the gene deletions by PCR. Mostly, three independent strains with a gene deletion (designated Δ1, Δ2, and Δ3) were used in PCR along with their wild-type (WT) progenitor and those carrying the transgene at an ectopic position (E). The primer pairs (S15 Table) used for PCR amplification are indicated below the gels.
(PDF)

S1 Table Genes differentially expressed in the *F*. *graminearum* strains lacking the *MAT*-deletions, or overexpressing *MAT1-2-1*, compared to the expression levels in the wild-type (WT) Z3643 strain. (XLSX)

S2 Table Genes differentially expressed in the *F*. *graminearum* strains lacking the *MAT*-deletions, compared to the expression levels in the *MAT-*null (Δ*MAT1-1*; Δ*MAT1-2*) strain. (XLSX)

S3 Table Gene ontology analysis of DEGs identified in this study (XLSX)

S4 Table Genes whose protein products are known to be involved in sexual development in other model organisms, which are not enriched for GO-terms (XLSX)

S5 Table DEGs whose gene expressions in the *MAT*-deletion strains were further determined by qPCR analysis (XLSX)

S6 Table Putative secondary metabolite(SM) gene clusters found among the DEGs identified in this study (XLSX)

S7 Table DEGs overlapped with genes differentially expressed during the perithecium formation, identified by Hallen et al [22] (XLSX)

S8 Table DEGs overlapping between Δ*FgVelB* and Δ*GzGPA1* strains [27, 28]. (XLSX)

S9 Table DEGs overlapping between Δ*SmtA-1*, Δ*SmtA-2*, and Δ*Smta-1* of *Sodaria macrospora* [15, 19]. (XLSX)

S10 Table Numbers of the DEGs under investigation for their functions (XLSX)

S11 Table Molecular characterization of the selected DEGs identified in this study (XLSX)

S12 Table Developmental time-course expression of the selected DEGs during sexual development, determined by qPCR (XLSX)

S13 Table DEGs overlapping with the transcriptomics data during sexual developmental processes, generated by Sikhakolli et al. [23]. (XLSX)

S14 Table Expression of genes involved in signal transduction pathways in the *F*. *graminearum MAT*-deletion strains, determined by qPCR (XLSX)

S15 Table Primers used in this study (XLSX)

S16 Table DEGs carrying the core consensus binding motifs of MAT1-2-1 in their promoter regions (XLSX)
