# Supplementary material for: A Large-Scale Functional Analysis of Putative Target Genes of Mating-Type Loci Provides Insight into the Regulation of Sexual Development of the Cereal Pathogen Fusarium graminearum
Source: PLoS Genet. 2015 Sep 3;11(9):e1005486. doi: 10.1371/journal.pgen.1005486 (PMC4559316; doi:10.1371/journal.pgen.1005486)
Supplement: S1 Text — (DOCX) [file pgen.1005486.s001.docx]

**S1 text**

**Targeted deletion and overexpression of *MAT* genes**

For the microarrays, we used four transgenic strains that were derived from the self-fertile wild-type (WT) strain (Z3643). Three strains, designated Δ*MAT1-1*, Δ*MAT1-2*, and Δ*MAT1-1*;Δ*MAT1-2*, contained different deletions of the two *MAT* loci (*MAT1-1*, *MAT1-2*), and one strain (OM2) overexpressed the *MAT1-2-1* allele. The Δ*MAT1-1* and Δ*MAT1-2* strains were constructed by simultaneously deleting three *MAT* transcripts (*MAT1-1-1*, *MAT1-1-2*, *MAT1-1-3*) located at the *MAT1-1* locus, or by deleting a single transcript (*MAT1-2-1*) at the *MAT1-2* locus, respectively [[11](#_ENREF_11)]. The entire region containing both *MAT1-1* and *MAT1-2* loci was deleted in Δ*MAT1-1*;Δ*MAT1-2* (S1 Fig.). The OM2 strain was generated by introducing an intact copy of *MAT1-2-1* under the control of a strong fungal promoter (from the *F. fujikuroi EF1A* gene) into the genome of Δ*MAT1-2* (S2 Fig). Unlike Z3643, all of the three *MAT-*deletion strains were self-sterile on carrot agar. OM2 was comparable to Z3643 in all of the mycological traits examined, including sexual development (S3 Fig.).

## Generation of *MAT-*null (T43ΔM1M2) and *MAT1-2-1-*overexpressing (OM2) strains

## The DNA constructs for deleting the regions carrying the ORFs at the *MAT1-1* and *MAT1-2* loci from the genome of *F*. *graminearum* strain Z3643 were created using double joint (DJ)-PCR [[1^*^](#_ENREF_68)]. Both DNA fragments (~1.0 kb each), corresponding to regions 5′- to the *MAT1-2-1* ORF and 3′- to the *MAT1-1-3* ORF, were amplified from Z3643 using primer pairs MATlarge5f /Matlarge5rt and MATlarge3ft/MATlarege3r, respectively (S15 Table). A 2.0kb fragment of the *hygB* (hygromycin B resistance gene) cassette was amplified from the plasmid pBCATPH using the primers HygB-for and HygB-rev. These three amplicons were mixed at a 1:2:1 molar ratio, and used as the template for a second round of PCR without primers. A final DJ-PCR product was amplified from the second PCR product using the nested primer pairs (MATlarge5N and MATlarge3N). A fused product of the *MAT1-2-1* ORF under control of the *F*. *fujikuroi* translation elongation factor gene (*EF1A*) promoter was amplified using a single joint-PCR using the primer pairs SK2707for/SK2707rev and MATSK2707tail/MATrev, as well as the nested primer pair 0.7kb-1.1kp and was then cloned into the pGEMT vector (Promega, Madison, WI, USA). These transforming constructs, for generation of the *MAT-*null and OM2 strains, respectively, were either added directly into fungal protoplasts alone or with pII99 [2^*^], respectively, for transformation as previously described [3^*^].

## A transforming DNA construct for gene deletion was integrated into the genome of Z3643 using a double crossover, which resulted in a *MAT-*null *F*. *graminearum* Z3643 strain (designated T43ΔM1M2; S1 Fig.). The targeted gene deletion was verified using DNA blot hybridizations (S1 Fig.). To generate transgenic *F*. *graminearum* strains overexpressing *MAT1-2-1*, the T43ΔM2-2 strain was complemented with an intact copy of the *MAT1-2-1* ORF under the control of a strong promoter from the *F .fujikuroi EFIA* gene, as described above, to generate the OM2 strain (S2A Fig.). Insertion of the transforming construct into the genome of T43ΔM2-2 was confirmed using DNA gel blot hybridization (S2B Fig.). The constitutive expression of *MAT1-2-1* in OM2 during both vegetative and perithecial induction conditions was confirmed using RT-PCR (S2C Fig.).

**References not cited in the manuscript**

1^*^. Yu JH, Hamari Z, Han KH, Seo JA, Reyes-Dominguez Y, Scazzocchio C. Double-joint PCR: a PCR-based molecular tool for gene manipulations in filamentous fungi. Fungal genetics and biology : FG & B. 2004;41(11):973-81. Epub 2004/10/07. doi: 10.1016/j.fgb.2004.08.001. PubMed PMID: 15465386.

2^*^. Namiki F, Matsunaga M, Okuda M, Inoue I, Nishi K, Fujita Y, et al. Mutation of an arginine biosynthesis gene causes reduced pathogenicity in Fusarium oxysporum f. sp. melonis. Molecular plant-microbe interactions : MPMI. 2001;14(4):580-4. Epub 2001/04/20. doi: 10.1094/MPMI.2001.14.4.580. PubMed PMID: 11310747.

3^*^. Lee T, Han YK, Kim KH, Yun SH, Lee YW. Tri13 and Tri7 determine deoxynivalenol- and nivalenol-producing chemotypes of Gibberella zeae. Applied and environmental microbiology. 2002;68(5):2148-54. Epub 2002/04/27. PubMed PMID: 11976083; PubMed Central PMCID: PMC127587.
