# Supplementary material for: A Large-Scale Functional Analysis of Putative Target Genes of Mating-Type Loci Provides Insight into the Regulation of Sexual Development of the Cereal Pathogen Fusarium graminearum
Source: PLoS Genet. 2015 Sep 3;11(9):e1005486. doi: 10.1371/journal.pgen.1005486 (PMC4559316; doi:10.1371/journal.pgen.1005486)
Supplement: S2 Text — (DOCX) [file pgen.1005486.s002.docx]

**Identification of a putative binding site for MAT1-2-1 protein using protein binding microarrays (PBM) and Electrophoretic mobility shift assay (EMSA)**

**Materials and Methods**

**PBM analysis**

Two different PBMs (Quadruple 9-mer-Based PBM [Q9-PBM] and FgPBM) were designed as previously described [[34](#_ENREF_40)]. The target probes in Q9-PBM were designed as follows. All possible combinations of 9-mer oligonucleotides were quadrupled, and a primer binding sequence and five thymidine linkers were attached to the slide. A total of 232,145 ( = 4^9^) probe features, including 131,072 features from all possible 9-mers and 101,073 replicated features, were designed. These repetitive sequences provided highly consistent results from which the consensus binding motifs could be extracted, thereby allowing unequivocal interpretation. FgPBM is another PBM assay used, which was specific to the natural promoter regions of *F*. *graminearum*. FgPBM consists of a total of 653,858 probes that were designed from 13,381 *F*. *graminearum* promoter sequences containing 40 bp long probes with 20 bp overlap (covering 1kb from 5′-upstream). The microarray chips were manufactured by Agilent, and the reverse complementary DNA strand of each probe was synthesized on the slide using thermo-stable DNA polymerase.

The partial cDNA sequence of *MAT1-2-1*, which encodes the 75 amino acid-HMG-box motif (5′-CGTATCCCACGACCTCCCAACGCTTACATCCTCTACCGCAAGGAGCGTCACCAGATCGTCAAGGGAAAGCGCCCTGGTATCACCAACAACGAGATTTCTCAAGTTCTTGGACGATGCTGGAACATGGAGCATCCTGACATTCGCACCTACTACAAGAAGATGGCAGACGACATTAAGGAGGAGCACAAGCGCCTGTACCCTGATTACCAGTACCGCCCTCGCAAG-3′) was cloned into the pET32(a) expression vector followed by the red fluorescent protein (DsRed) gene, and then expressed in *E*. *coli* BL21-CodonPlus (Stratagene, La Jolla, CA, USA). DsRed fluoresces at a similar spectrum to Cy3, and is compatible with the microarray scanner. The purified DsRed fusion protein was applied to double-stranded Q9-PBM and FgPBM, as previously described [[34](#_ENREF_40)], and the fluorescence intensity of the bound protein was acquired using a microarray scanner. The consensus binding sequence was determined based on signal strength. Generally, the rank-ordered signal distribution of the bound protein showed a deep leftward slope followed by a heavy right tail (S7 Fig.), consistent with a previous report [[35](#_ENREF_41)]. Two independent linear models, y = ax + b, were applied to the deep and the heavy right tail region using R statistical language. Spots that exhibited strong intensity and high enrichment were then aligned.

**EMSA**

EMSA was performed as previously described [[34](#_ENREF_40)]. Biotin end-labeled and -unlabeled oligonucleotides (Bioneer) were annealed to each complimentary sequence. A total of 5 µg MAT1-2-1 protein was incubated with 40 fmol biotin-labeled double-stranded oligonucleotides, 1 µg poly dI-dC, 1× binding buffer, 2.5% glycerol, and 0.05% NP40 in a 20 µl reaction volume for 30 min at room temperature, according to the manufacturer’s instructions (Pierce, Rockford, IL, USA). The reaction mixture was analyzed using electrophoresis on a non-denaturing 6% polyacrylamide gel with 0.5× TBE buffer. The DNA-protein complexes in the gel were electrophoretically transferred to a positively charged nylon membrane in 0.5× TBE at 380 mA for 30 min, and then cross-linked at 120 mJ/cDMAT1-2 using a UV-light crosslinker. Biotin was detected using a Lightshif^TM^ Chemiluminescent EMSA kit (Pierce).

**Results**

**PBM and EMSA analyses**

Next, we used PBM technology [34, 35] (see the Materials and Methods for details) to identify a putative binding site for the MAT1-2-1 protein. Two PBMs (Q9-PBM, FgPBM) were hybridized to the DNA-binding HMG motif of MAT1-2-1, which was fused to DsRed fluorescent protein, and then expressed in *E*. *coli*. The consensus binding sequence was determined based on signal strength. The rank-ordered signal distribution of the bound protein exhibited a deep leftward slope, followed by a heavy right tail (S7 Fig.), consistent with a previous report [34]. Because the probes in the deep slope region differed by only one base, we assumed that the signal distribution was due to a specific interaction between the protein and features on the microarray. The spot intensities were rank-ordered, and the enrichment scores were determined. Spots that exhibited a strong intensity and high enrichment were then aligned. Q9-PBM analysis identified three clusters (ACTACAGAA, ATTGTTCCA, ATTAATATC) as putative consensus binding motifs, among which ATTGTTCCA had the highest frequency (Fig 7). The position matrix of the ATTAAT motif is shown in S8 Fig.

**Fig. 7. The determined consensus binding sequences according to the PBM results.** To determine binding motifs, two independent linear models were applied in the deep and the heavy right tail region.

A total of 4,850 genes in the *F*. *graminearum* genome carried the ATTAAT motif in their putative promoter region (1,000 bp upstream of the start site of each ORF), among which 163 genes were downregulated and 107 were upregulated in *MAT*-deletion strains (S16 Table). The total number of DEGs (270) was identical to the distribution expected from the chance by which the ATTAAT motif could occur in the DEG promoter regions (1,106 × 1,000 / 4^6^ = 270), suggesting that the ATTAAT motif is not significantly enriched in the DEG promoter regions. The frequency of this motif (42/177 = 23.7%) among the downregulated genes in OM2 (S16 Table) was not significantly different from what was expected (270/1,106 = 24.4%). However, ~50% of the DEGs (68 of 135 the downregulated genes; 45 of 92 upregulated genes) carrying the ATTAAT motif were involved in metabolism, including cell wall biosynthesis. In addition, DEGs carrying the HMG motif included regulatory genes such as two TFs (FGSG_01366, FGSG_05151), signal transduction pathway components, those involved in cytoskeleton dynamics (e.g., kinetochore, kinesin, actin polymerization), and membrane proteins (S16 Table). Interestingly, the ATTAAT motif was also present in the putative promoter region of *MAT1-2-1* (S16 Table).

FgPBM analysis revealed a specific interaction between probes of the natural promoter and MAT1-2-1, which was also confirmed by the rank-ordered signal distribution as shown in Q9-PBM (rank expolarated is 2,076) (S7 Fig.). Four putative binding motifs (ATTGTTTTG, AAATGAAAA, AATTGATGA, ATCATCAAT) were identified, among which ATTGTTTTG was the most significant binding motif based on the Wilcox-Mann P-value analysis. The position weight matrix of ATTGTTTTG is shown in S8 Fig. This motif was identified in the putative promoter regions of 644 genes in the *F*. *graminearum* genome, among which 75 (11.6%) overlapped with DEGs in the *MAT* deletion and *MAT1-2-1-*overexpressing strains (S16 Table). Of these, *MAT1-1-3*, 2 TFs (FGSG_05151, FGSG_09019) carrying the HMG motif and homeodomain, respectively, and 32 genes possibly involved in metabolism were identified. In addition, 21 genes contained both the ATTAAT and ATTGTTTTG motifs in their promoter regions, including a TF carrying the HMG-motif (FGSG_05151) (S16 Table). A comparison of the putative consensus binding sequences identified using both PBM methods revealed that ATTGTT could be the core binding sequence for the HMG domain of MAT1-2-1 because it was present in both PBM analyses.

To confirm the binding activity of the consensus sequences, we selected the six base pair (bp) candidate sequences ATTAAT and ATTGTT, and then performed an electrophoretic mobility shift assay (EMSA). The quadruple sequences of these motifs showed clear binding activity for the MAT1-2-1 protein, but the interaction with ATTGTT was weaker than that with ATTAAT (S9A Fig.). To assess the binding activity of natural promoter sequences (each 20 bp long) that contain the consensus sequences, EMSAs were performed using 20 bp sequences of the putative promoter regions of 16 genes that were selected based on spot intensities in PBM analyses and their possible functions. Data revealed that the promoter region of FGSG_04946, which carries the ATTGTT motif in reverse, could bind to MAT1-2-1. Two genes (FGSG_08467 and FGSG_06480), both of which also contained the ATTGTT motif, exhibited weaker binding activity (S9B Fig.). However, only one gene (FGSG_04946) was a DEG (DNN-type) identified in the DNA microarray analysis (S11 Table).

**Discussion**

The putative DNA binding sites of MAT1-2-1 protein identified using a PBM assay in the current study is significant because i) it provides the first evidence for the MAT1-2-1 HMG box-specific binding sequence, which was identified using a genome-wide search, and ii) the core binding site (ATTGTT) for the MAT1-2-1 HMG domain identified here is complementary to the core-binding element (AACAAT) of the mammalian sex-determining region Y (*SRY*) or *SRY*-related HMG box gene (*SOX*) [[36-38](#_ENREF_36)]. Regarding the first significant finding, the DNA binding sequences of the HMG motif-containing MAT protein were only analyzed in *Schizosaccharomyces pombe* and *N*. *crassa* using previously characterized HMG box binding motifs [[48](#_ENREF_48)] or restricted DNA regions (*MAT* locus) [1^*^]. Except for the MAT proteins in yeast and *N*. *crassa*, the *in vitro* DNA binding property of the HMG motif-containing MAT protein has not been elucidated in filamentous ascomycetes, probably due to the insolubility of full-length MAT proteins such as MAT1-2-1 [this study] and MT a-1 [1^*^] expressed in *E*. *coli*. However, the full-length MAT1-1-1 protein carrying a DNA-binding alpha domain from *P. chrysogenum* was expressed in *E. coli* and successfully used in ChIP-seq analysis for the identification of target genes for MAT1-1-1 [[21](#_ENREF_21)]. Here, we circumvented this problem by expressing only the HMG motif region in *E*. *coli*, since it is known that the HMG box domain alone from the fungal mating type genes or mammalian *SOX* genes is sufficient for binding to DNA *in vitro* [1^*^-3^*^].

The consensus between the DNA binding motifs from MAT1-2-1 and mammalian SRY and SOX proteins could be evidence that supports the reliability of our PBM analysis. Although the putative DNA binding sites were not significantly enriched in the promoter regions of the DEGs identified in the current study, four TFs (MAT1-1-3, MAT1-2-1, FGSG_01366, FGSG_05151) carrying the HMG-box motif were enriched among the DEGs carrying the core sequences. This suggests that MAT1-2-1 protein might control the expression of these TFs, by directly binding to their promoters. Considering the sexual-specific phenotypes of deletion strains lacking these TFs, these HMG-box-containing TFs might be important targets of MAT1-2-1 to control the sexual developmental processes in *F*. *graminearum*. The EMSA results suggest that the MAT1-2-1 protein may have the ability to bind to core sequences *in vitro*. Weak interactions between the HMG motif of MAT1-2-1, and its putative target promoter sequence in the EMSA could be attributed to the absence of cofactors that might influence the DNA binding specificity and affinity of homeobox proteins [4^*^, 5^*^]. However, the weak DNA binding signals do not exclude the possibility that there is no specific binding of MAT1-2-1 to the putative target promoters.

As an alternative or additional strategy for the genome-wide identification of binding sites for MAT1-2-1 target genes, a ChIP seq technique could be used in *F. graminearum*. However, considering the time and effort required for ChIP seq and subsequent analyses, as shown in Becker et al’s (2015), it would be an independent task for another investigation.

**References not cited in the manuscript**

1^*^. Philley ML, Staben C. Functional analyses of the *Neurospora crassa MT a-1* mating type polypeptide. Genetics. 1994;137: 715-22.

2^*^. Dooijes D, van de Wetering M, Knippels L, Clevers H. The *Schizosaccharomyces pombe* mating-type gene *mat-Mc* encodes a sequence-specific DNA-binding high mobility group box protein. J Biol Chem. 1993;268: 24813-7.

3^*^. Mertin S, McDowall SG, Harley VR. The DNA-binding specificity of SOX9 and other SOX proteins. Nucleic Acids Res 1999;27: 1359-64.

4^*^. Gstaiger M, Georgiev O, van Leeuwen H, van der Vliet P, Schaffner W. The B cell coactivator Bob1 shows DNA sequence-dependent complex formation with Oct-1/Oct-2 factors, leading to differential promoter activation. EMBO J. 1996;15: 2781-90.

5^*^. Zhong H, Vershon AK. The yeast homeodomain protein MATalpha2 shows extended DNA binding specificity in complex with Mcm1. J Biol Chemi. 1997;272: 8402-9.
