# Supplementary figures and images for: A Large-Scale Functional Analysis of Putative Target Genes of Mating-Type Loci Provides Insight into the Regulation of Sexual Development of the Cereal Pathogen Fusarium graminearum
Source: PLoS Genet. 2015 Sep 3;11(9):e1005486. doi: 10.1371/journal.pgen.1005486 (PMC4559316; doi:10.1371/journal.pgen.1005486)

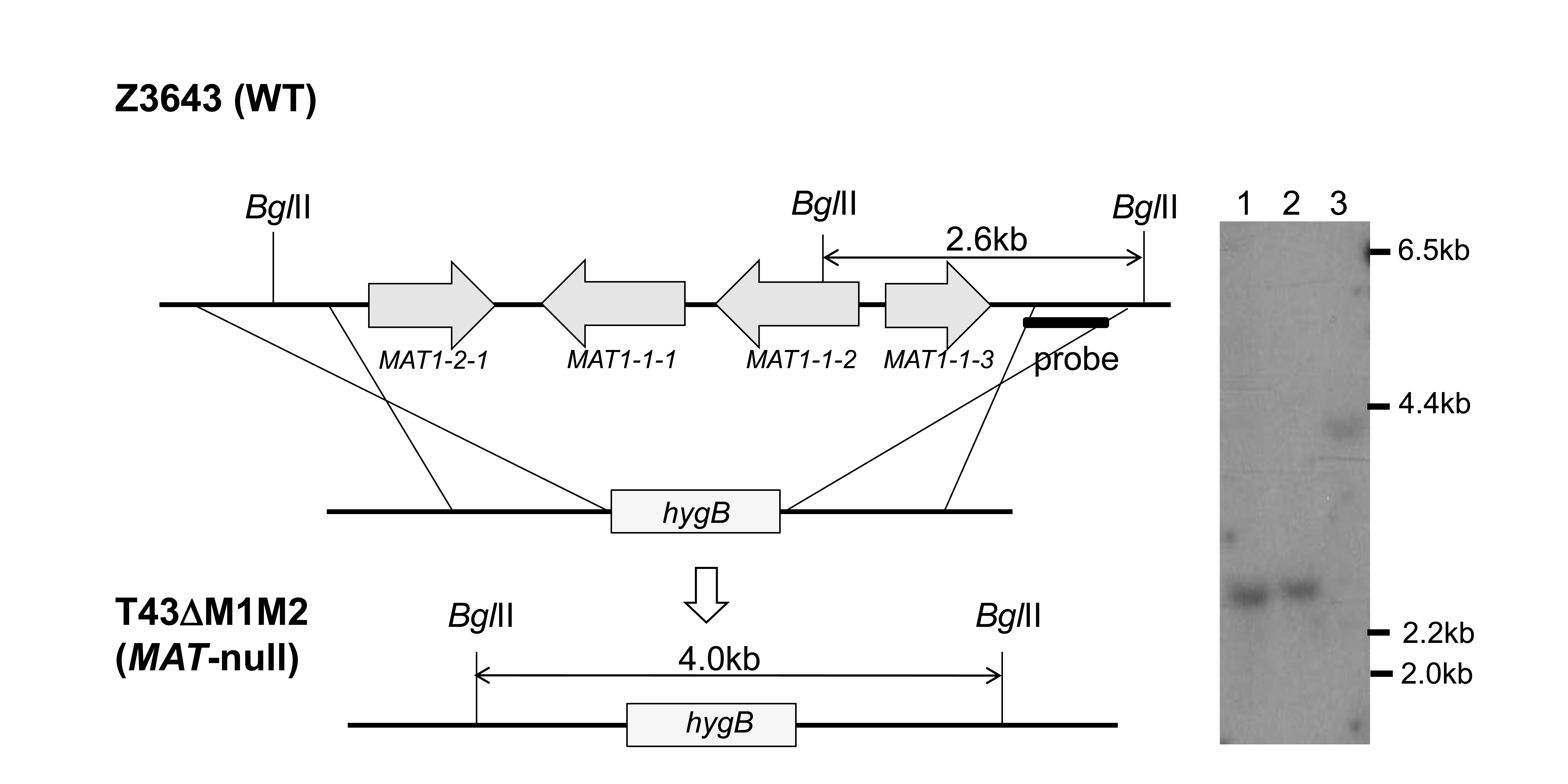

Supplement: S1 Fig — Left panel: deletion scheme. Z3643 (WT), genomic DNA of the F. graminearum wild-type Z3643 strain; T43ΔM1M2 (MAT-null), genomic DNA of a transgenic Z3643 strain lacking the entire MAT loci. Right: BglII-digested genomic DNA gel blot hybridized with the probe indicated in the deletion scheme. Lanes 1 and 2, Z3643 (WT); 3, T43ΔM1M2. DNA size markers are indicated on the right side of the gel. (TIF) [file pgen.1005486.s003.tif]

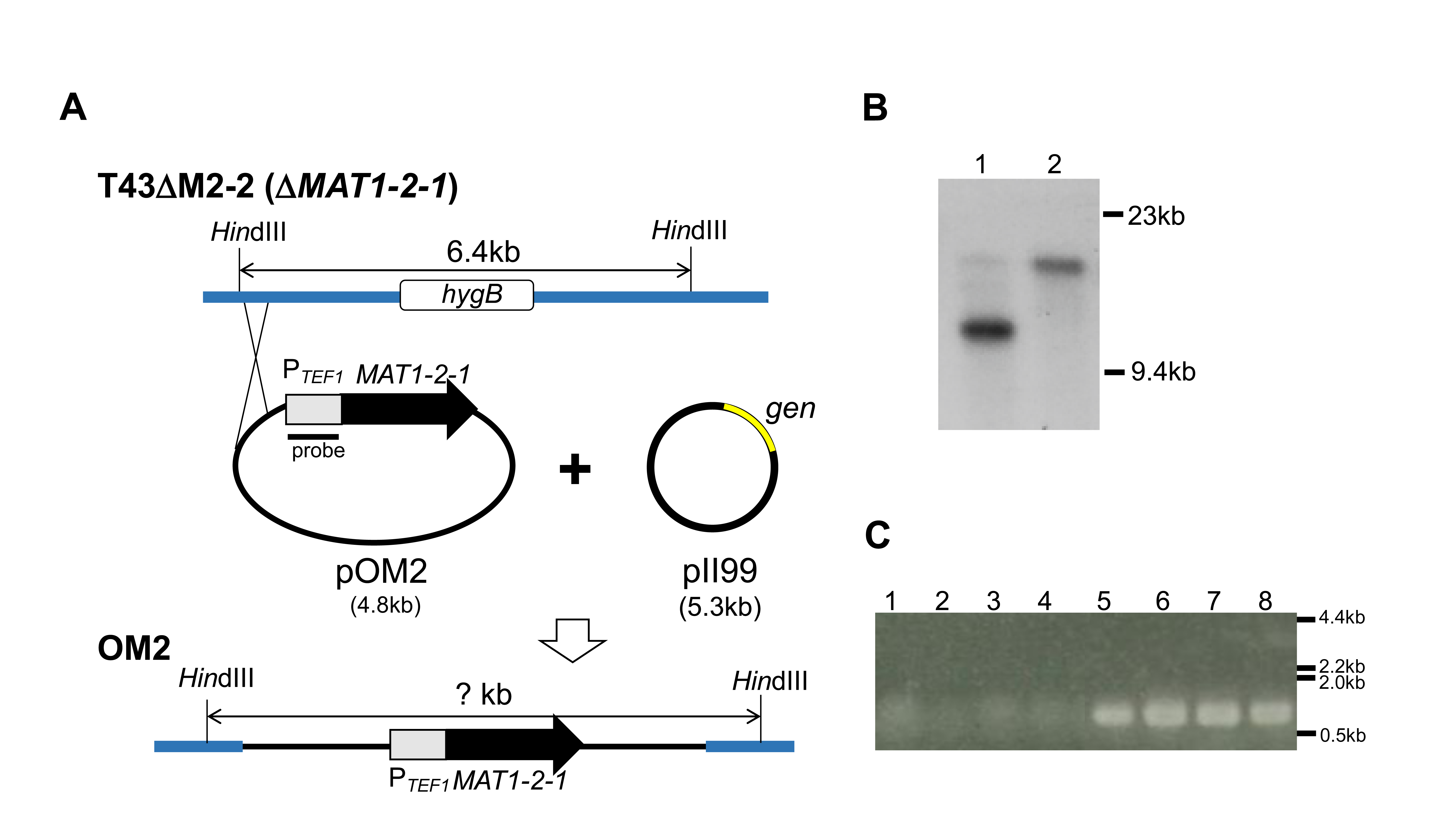

Supplement: S2 Fig — (A) The scheme for insertion of the pOM2 vector, which carries a MAT1-2-1 overexpressing construct, into the genome of a F. graminearum MAT1-2-1-deleted strain by a non-homologous gene integration event. Note that there is no homology between the pOM2 vector and ΔMAT1-2-1 genome. The geneticin resistance gene (gen) in pII99 vector was used as a selectable marker in a co-transformation strategy. T43ΔM2–2 (ΔMAT1-2-1), genomic DNA of a transgenic F. graminearum Z3643 strain where the MAT1-2-1 gene was replaced with the hygromycin B resistance gene (hygB); OM2, genomic DNA of a geneticin-resistant F. graminearum transformant carrying the MAT1-2-1 overexpressing construct. PTEF1: F. fujikuroi EF1A promoter region. (B) HindIII-digested DNA gel blot of the OM2 strains, hybridized with a probe amplified from PTEF1. Lanes 1 and 2, OM2 strains showing different-, but larger-sized hybridizing bands than 4.8 kb (pOM2) (due to ectopic vector insertions). (C) Amplification of MAT1-2-1 from total RNAs of the fungal strains by reverse transcription (RT) PCR. Lanes 1 and 2: RNA extracted from the F. graminearum wild-type Z3643 strain grown on carrot agar for 3 and 6 days, respectively (vegetative growth stage); lanes 3–4, those from the ΔMAT1-2-1 strain grown on carrot agar for 3 and 6 days after removal of aerial mycelia, respectively (perithecial induction stage); lanes 5–6, those from OM2 [from lane 2 in (B)] under vegetative growth conditions; lanes 7–8, those from OM2 under the perithecia induction stage. (TIF) [file pgen.1005486.s004.tif]

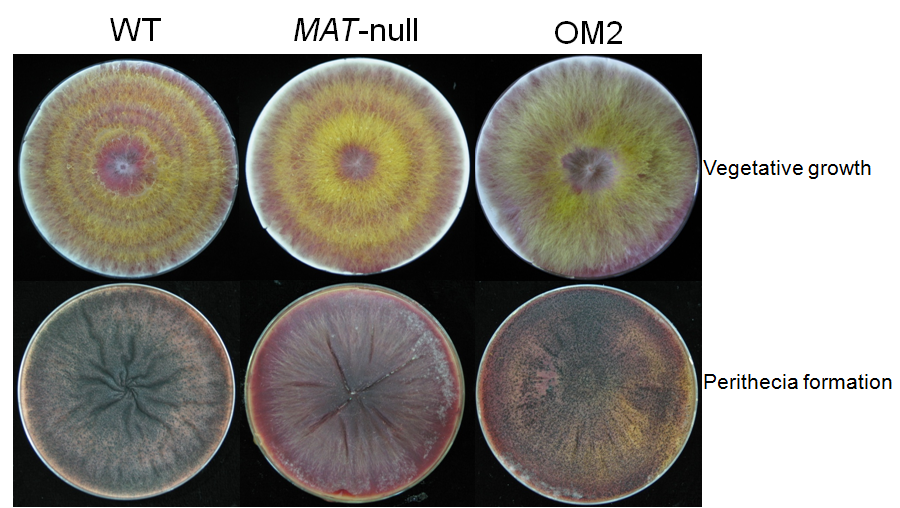

Supplement: S3 Fig — Vegetative growth, grown on carrot agar for 6 days; perithecia formation, grown on carrot agar 6 days after removal of aerial mycelia that had previously been grown for 6 days for vegetative growth. (TIF) [file pgen.1005486.s005.tif]

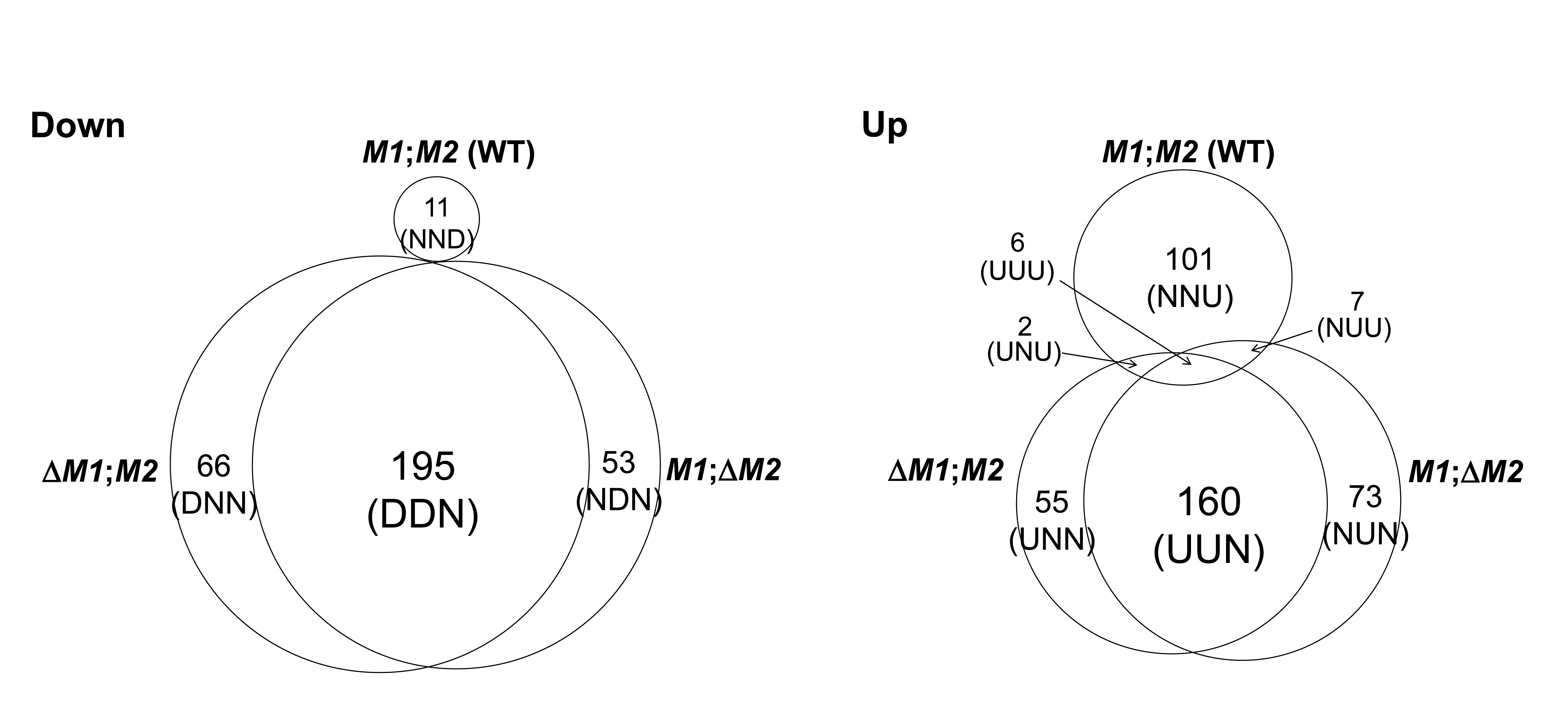

Supplement: S4 Fig — DOWN, down-regulated genes; UP, up-regulated genes. The numbers of differentially regulated genes in each expression category are indicated above the category designations in parentheses (e.g., “DDN” means downregulation in ΔMAT1-1, downregulation in ΔMAT1-2, and no change in ΔMAT1-1;ΔMAT1-2). (TIF) [file pgen.1005486.s006.tif]

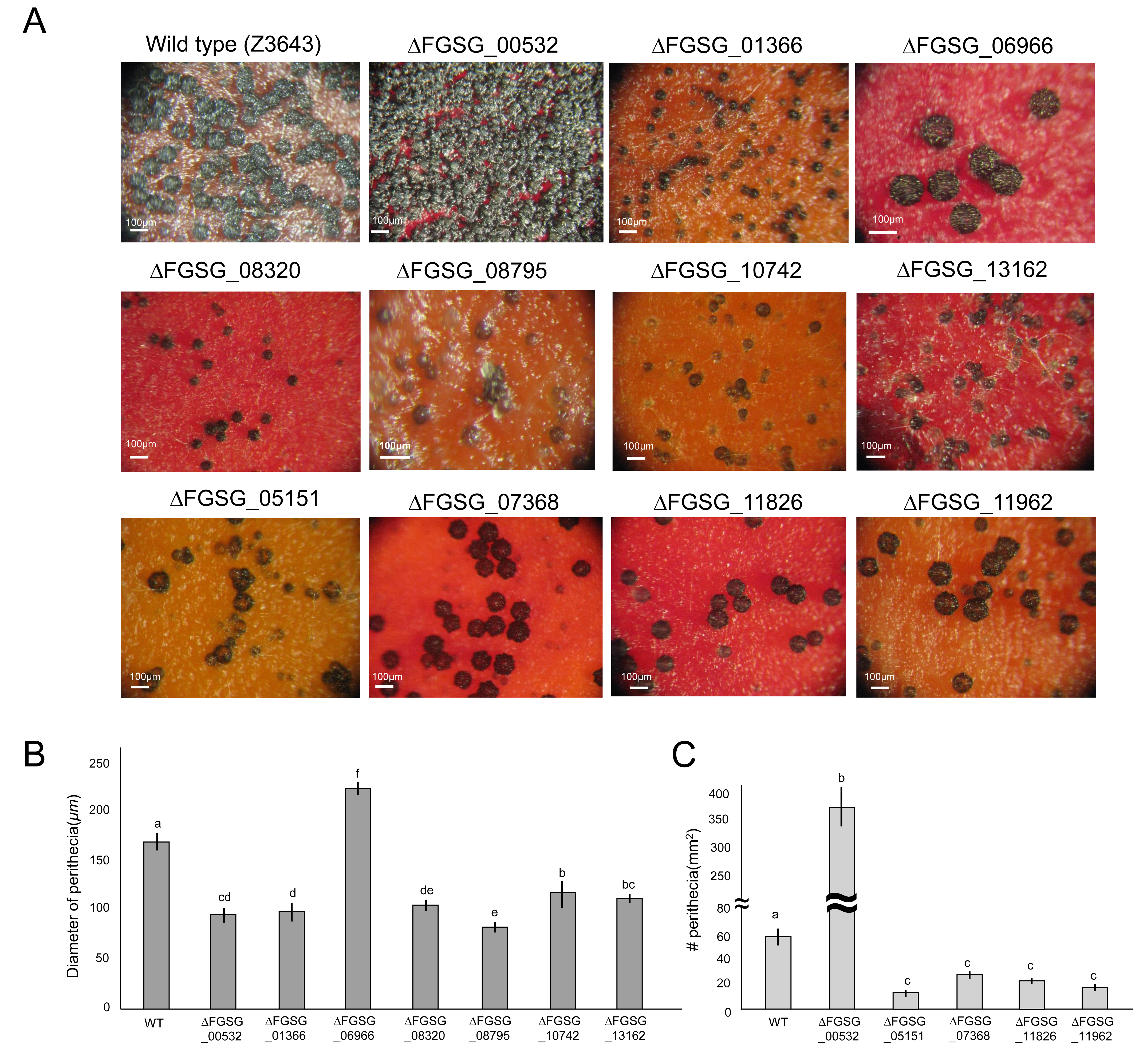

Supplement: S5 Fig — (A) Perithecia of the F. graminearum strains on carrot agar plates 6 days after perithecial induction. WT, a wild-type Z3643 strain; ΔFGSG_XXXX, a transgenic Z3643 strain deleted for the gene with the corresponding gene ID (FGSG). Scale bar = 100 μm. (B) Average diameters of the perithecia produced by the F. graminearum strains shown in A, which were calculated from the measurement of 100 perithecia for each strain under a dissecting microscope. (C) Average number of perithecia/mm2 on a carrot agar plate. The different letter above bars represent significant differences (p<0.05) based on Tukey’s test. (TIF) [file pgen.1005486.s007.tif]

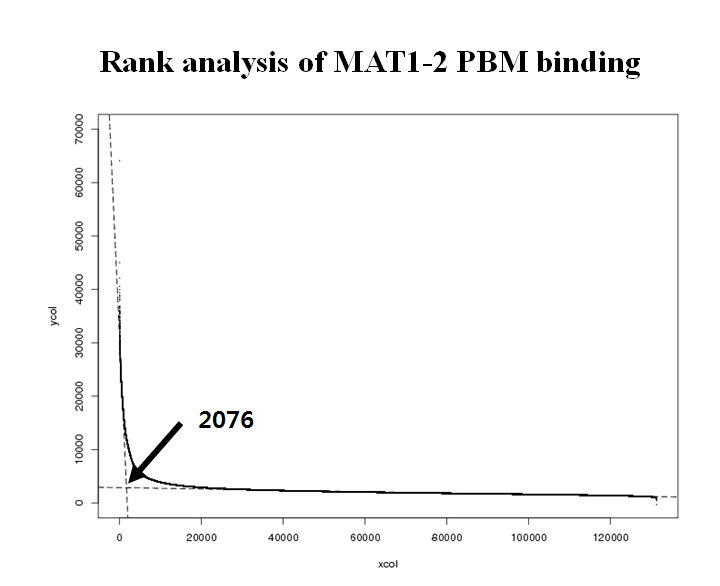

Supplement: S7 Fig — Two independent linear models, y = ax+b, were applied in the deep (b1 = 26656.3, slope = -28.3) and heavy right (b1 = 1.154e+03, slope = - -9.719e-03) tail regions. Rank extrapolated is 2076. (TIF) [file pgen.1005486.s009.tif]

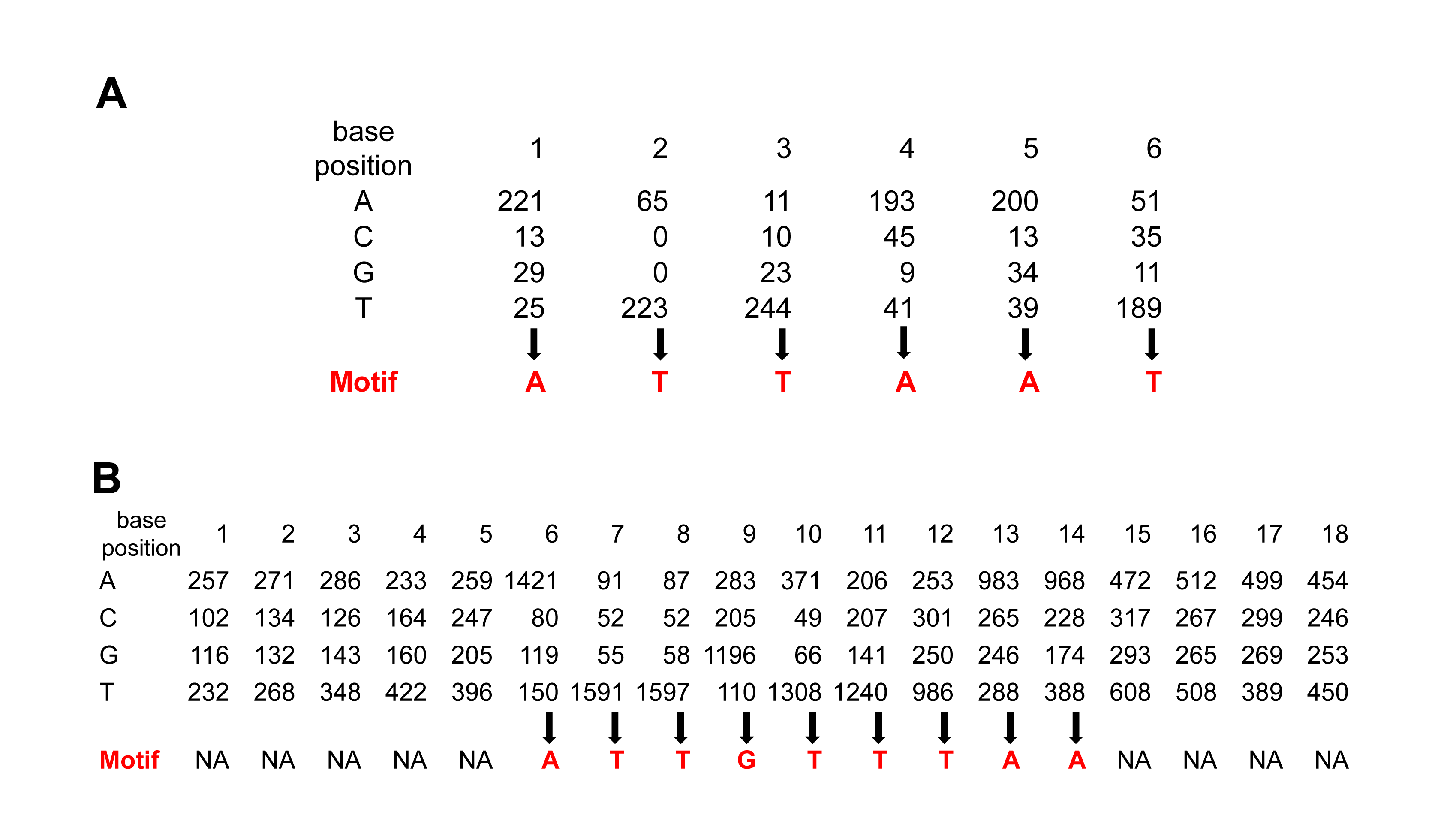

Supplement: S8 Fig — P-value for the motif enrichment is zero. (TIF) [file pgen.1005486.s010.tif]

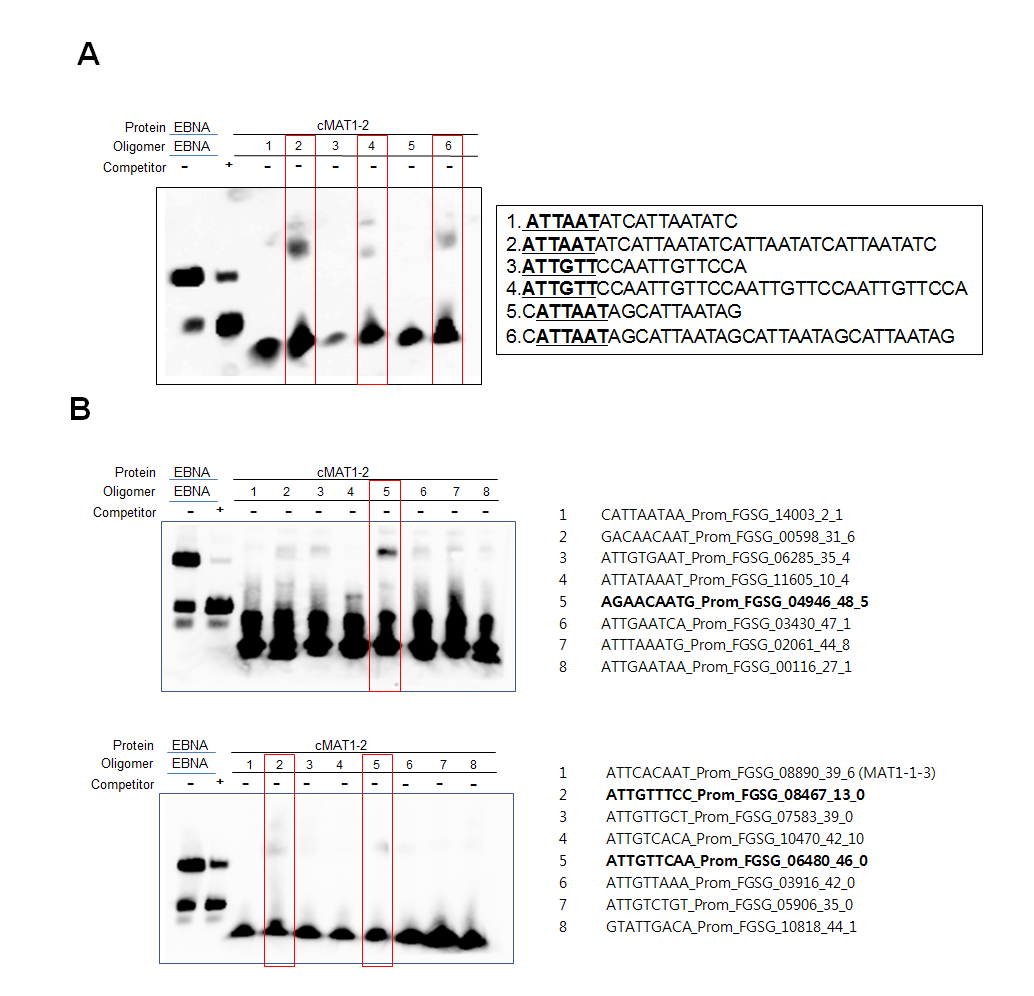

Supplement: S9 Fig — The duplicated or quadruple 9 bp sequences containing these motifs (A), or 20 bp sequences of the putative promoter regions containing the motifs from the selected 16 genes (designated with FGSG_ ID) (B) were used for the interaction. EBNA: the Epstein-Barr nuclear antigen control system, cMAT1-2: HMG motif of the F. graminearum MAT1-2-1 protein expressed in E. coli. (TIF) [file pgen.1005486.s011.tif]

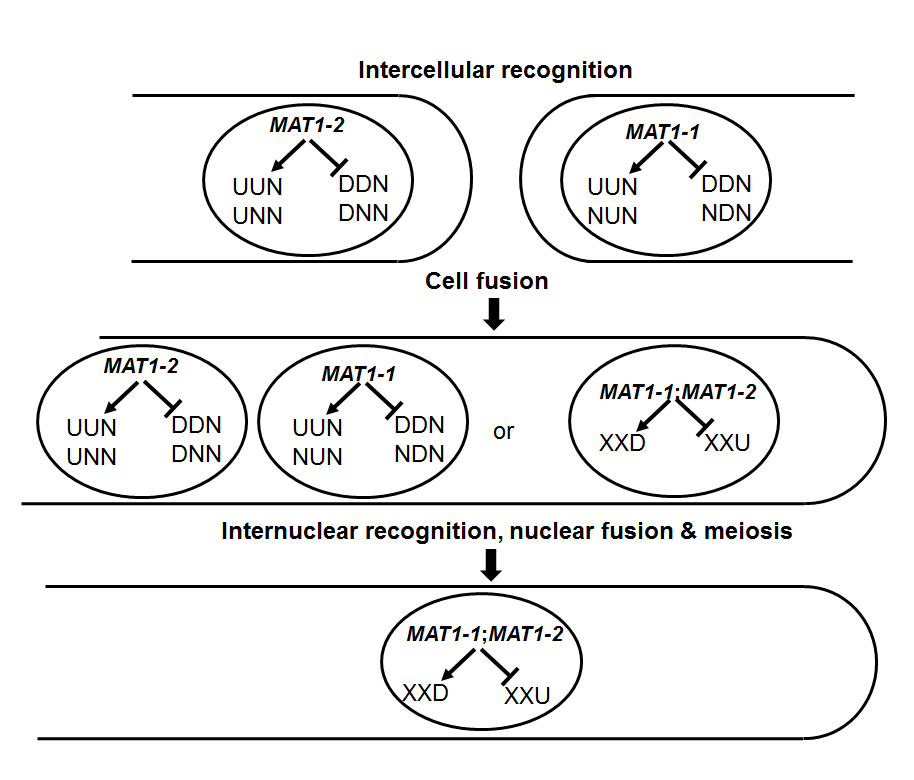

Supplement: S10 Fig — The single MAT genotype (either MAT1-1 or MAT1-2) of a fungal nucleus, depicted by a circle, is assumed based on the differential expression of both MAT loci. These nuclei may play a role in either intercellular or internuclei recognition. Both MAT loci, which could be expressed in both haploid and diploid cells, may control DEGs involved in meiosis and perithecia maturation. Abbreviations for differentially expressed genes with three characters: U, upregulated, D; downregulated; N, no change; X, either U or D. The first, second, and third characters represent the expression pattern in the ΔMAT1-1, ΔMAT1-2, and ΔMAT1-1;ΔMAT1-2 strains, respectively, compared to the Z3643 strain. Solid lines with triangular and flattened arrowheads represent gene activation and repression, respectively. (TIF) [file pgen.1005486.s012.tif]

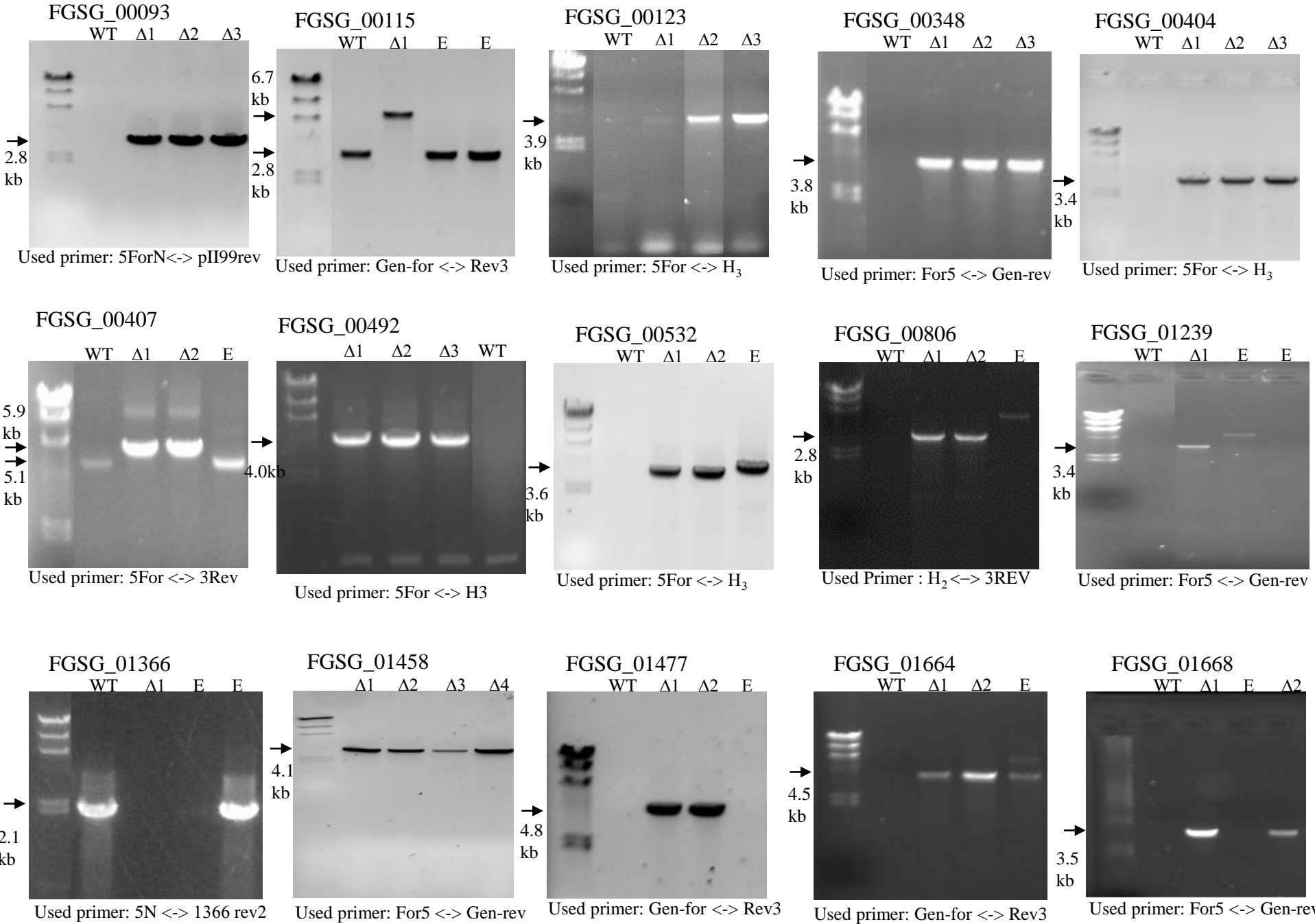

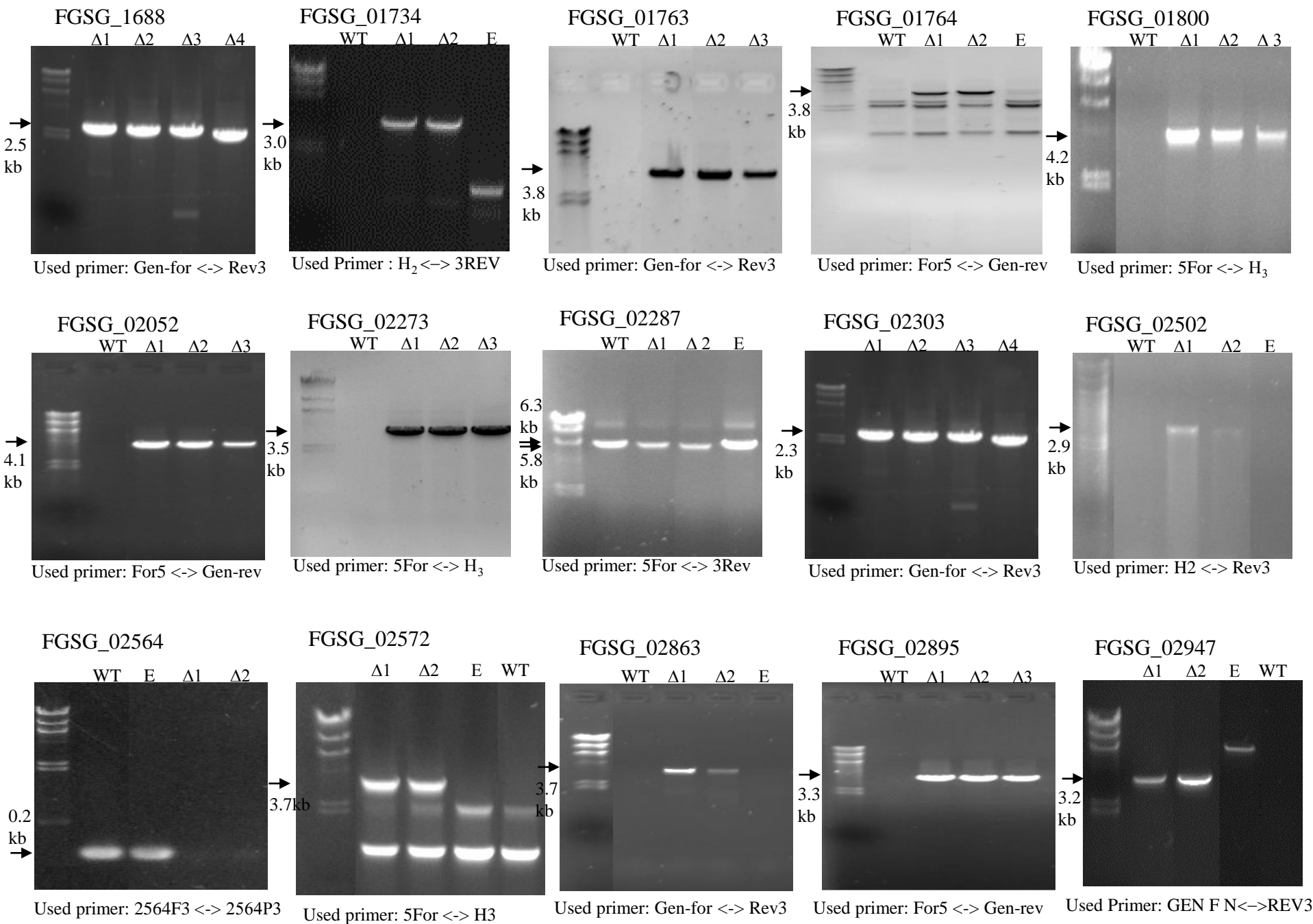

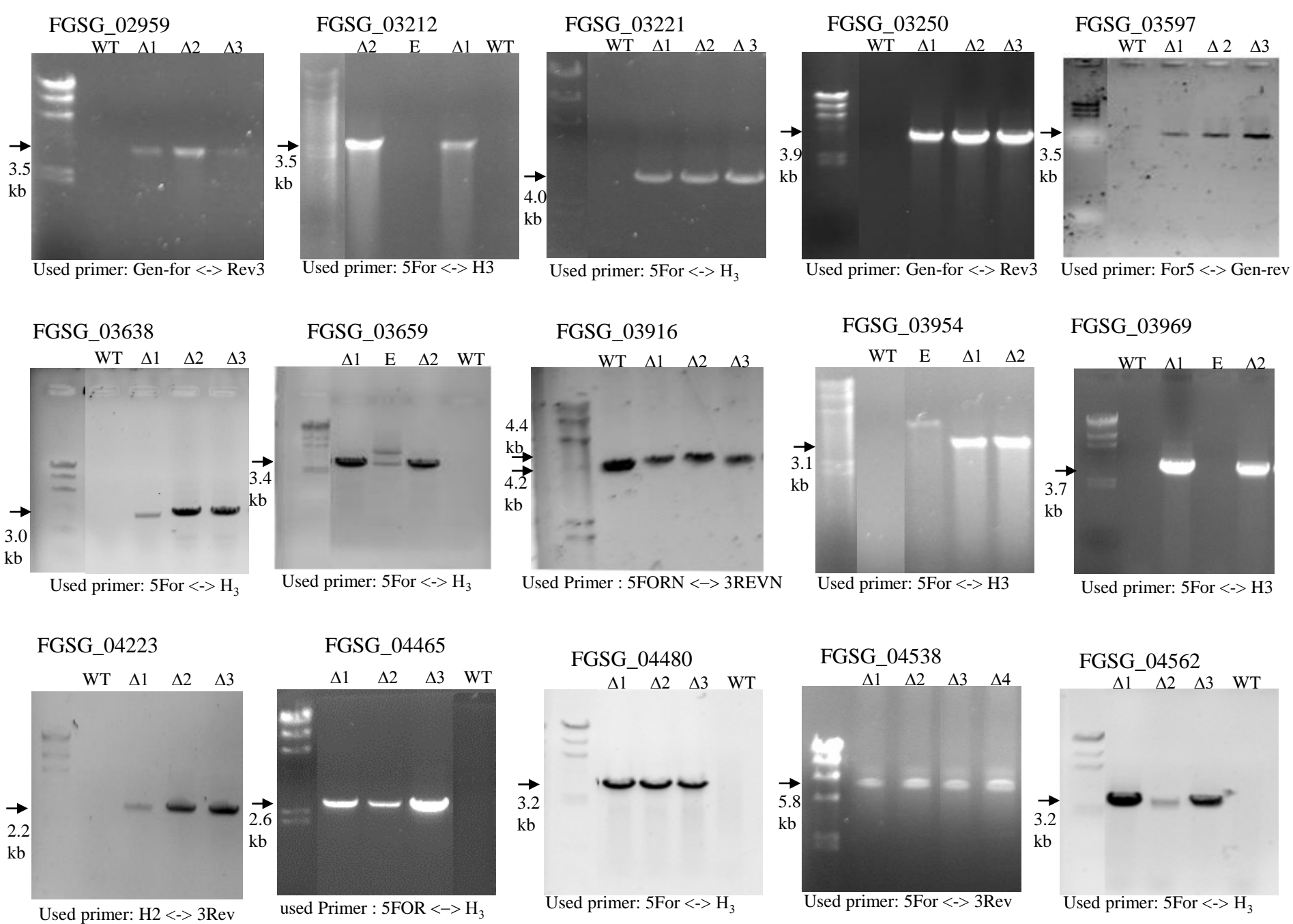

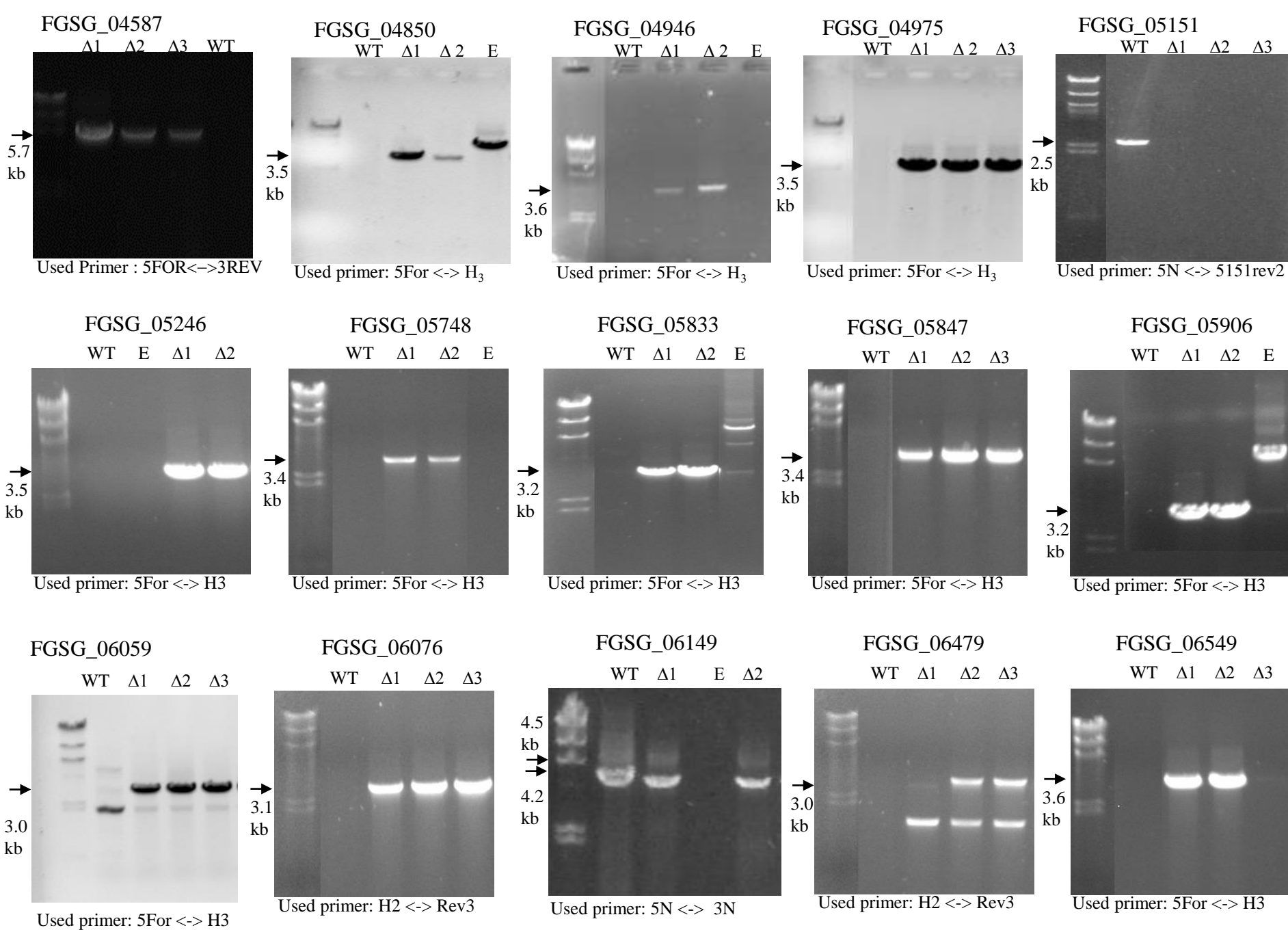

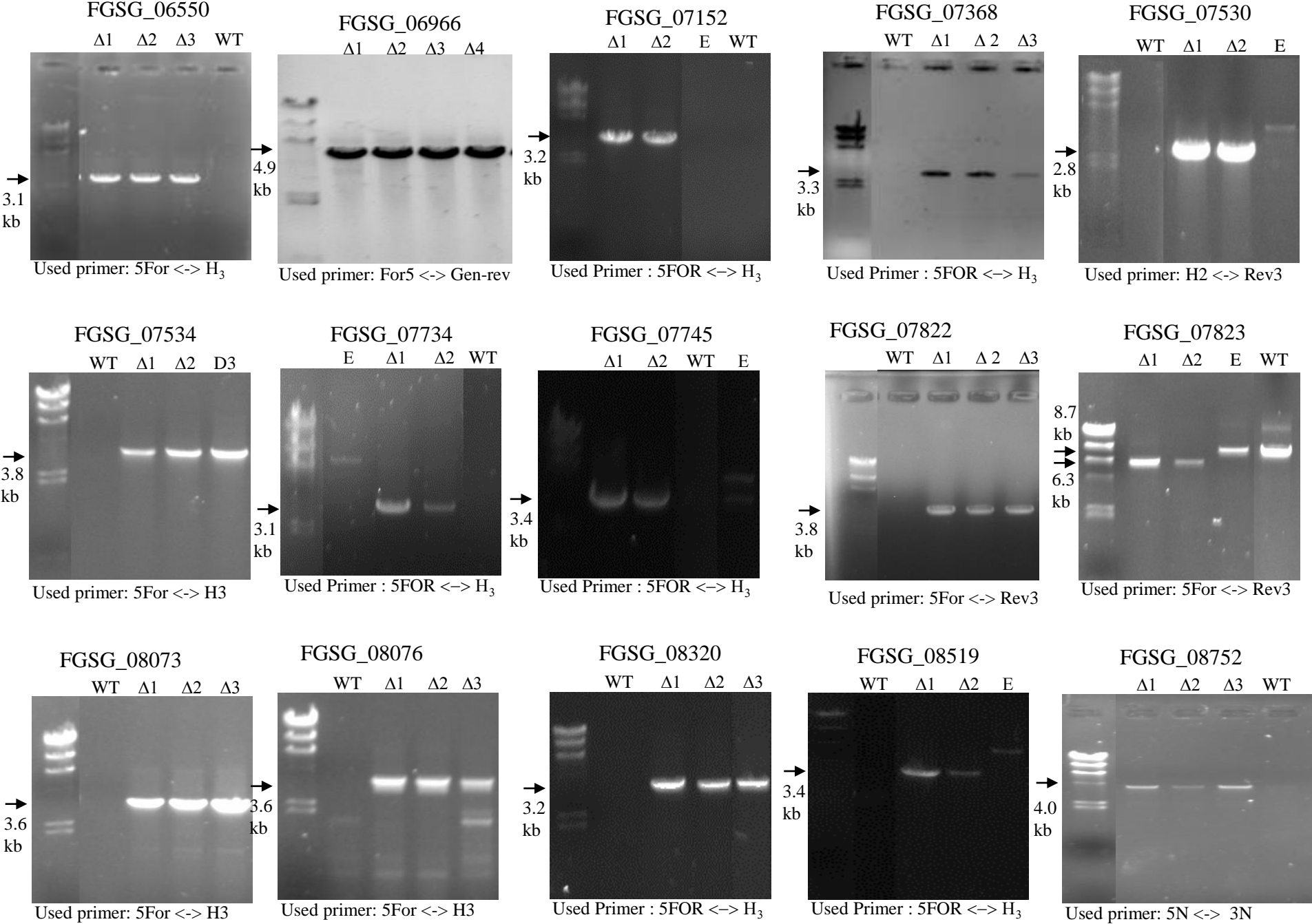

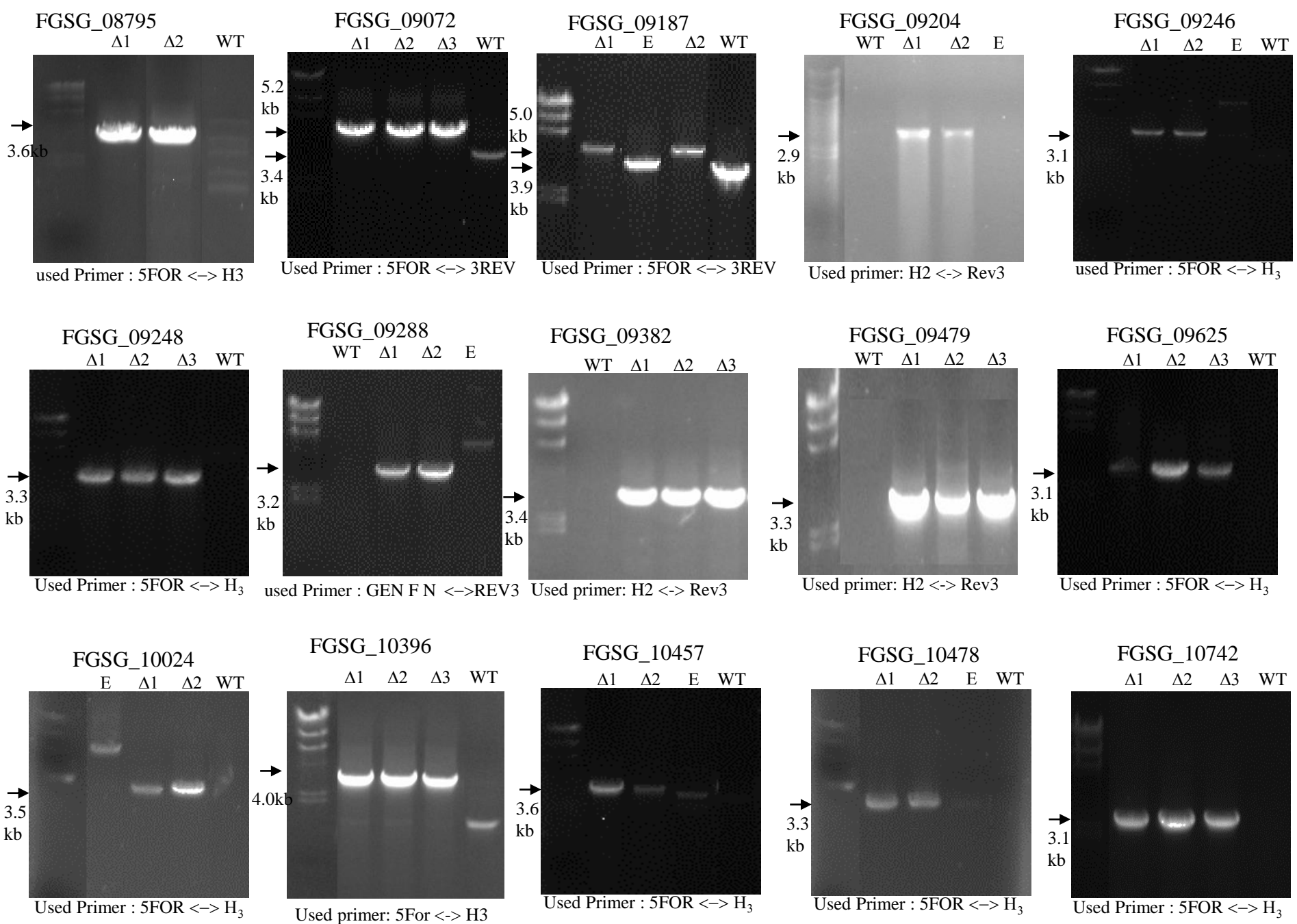

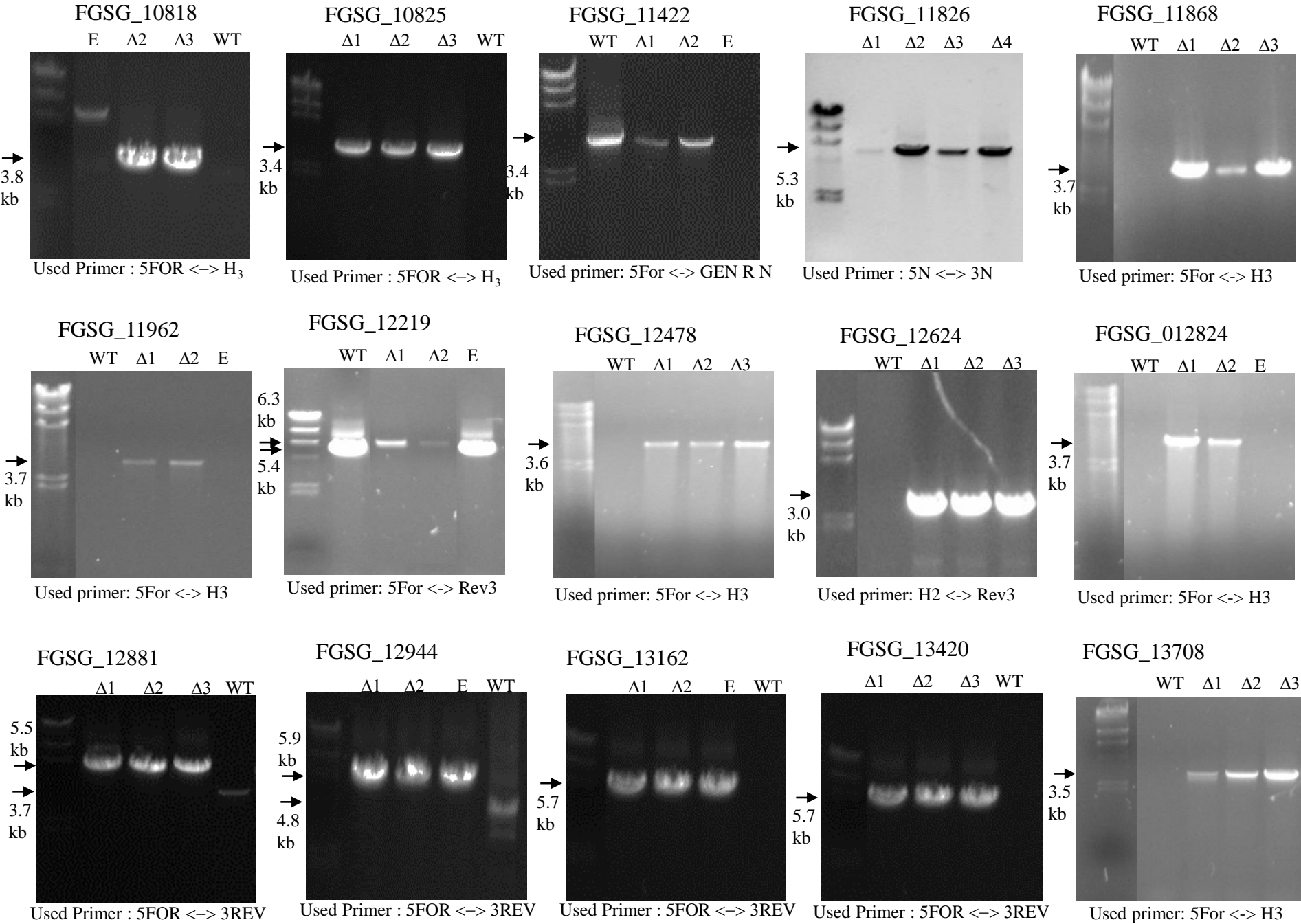

FGSG\_13860

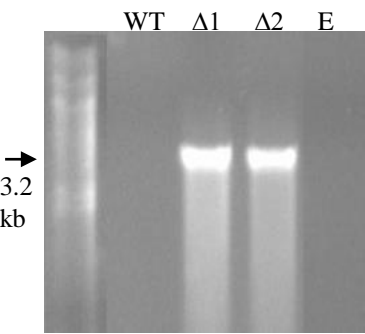

FGSG\_14031

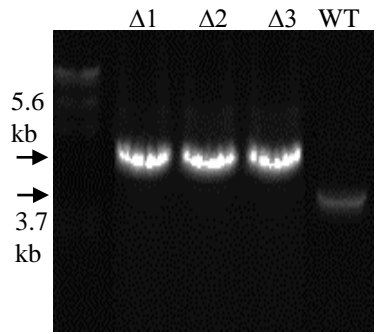

Supplement: S11 Fig — Mostly, three independent strains with a gene deletion (designated Δ1, Δ2, and Δ3) were used in PCR along with their wild-type (WT) progenitor and those carrying the transgene at an ectopic position (E). The primer pairs (S15 Table) used for PCR amplification are indicated below the gels. (PDF) [file pgen.1005486.s013.pdf]
